# A plant alkaloid, veratridine, potentiates cancer chemosensitivity by UBXN2A-dependent inhibition of an oncoprotein, mortalin-2

**DOI:** 10.18632/oncotarget.4452

**Published:** 2015-07-11

**Authors:** Ammara Abdullah, Sanam Sane, Kate A. Branick, Jessica L. Freeling, Hongmin Wang, Dong Zhang, Khosrow Rezvani

**Affiliations:** ^1^ Division of Basic Biomedical Sciences, Sanford School of Medicine, The University of South Dakota, Vermillion, SD 57069, USA; ^2^ Department of Biomedical Sciences, College of Osteopathic Medicine, New York Institute of Technology, Old Westbury, NY 11568, USA

**Keywords:** veratridine, UBXN2A, p53, mortalin-2, chemotherapy

## Abstract

Veratridine (VTD), an alkaloid derived from the Liliaceae plant shows anti-tumor effects; however, its molecular targets have not been thoroughly studied. Using a high-throughput drug screen, we found that VTD enhances transactivation of UBXN2A, resulting in upregulation of UBXN2A in the cytoplasm, where UBXN2A binds and inhibits the oncoprotein mortalin-2 (mot-2). VTD-treated cancer cells undergo cell death in UBXN2A- and mot-2-dependent manners. The cytotoxic function of VTD is grade-dependent, and the combined treatment with a sub-optimal dose of the standard chemotherapy, 5-Fluorouracil (5-FU) and etoposide, demonstrated a synergistic effect, resulting in higher therapeutic efficacy. VTD influences the CD44+ stem cells, possibly through UBXN2A-dependent inhibition of mot-2. The VTD-dependent expression of UBXN2A is a potential candidate for designing novel strategies for colon cancer treatment because: 1) In 50% of colon cancer patients, UBXN2A protein levels in tumor tissues are significantly lower than those in the adjacent normal tissues. 2) Cytoplasmic expression of the mot-2 protein is very low in non-cancerous cells; thus, VTD can produce tumor-specific toxicity while normal cells remain intact. 3) Finally, VTD or its modified analogs offer a valuable adjuvant chemotherapy strategy to improve the efficacy of 5-FU-based chemotherapy for colon cancer patients harboring WT-p53.

## INTRODUCTION

The heat shock protein mortalin-2 (mot-2) excludes wild-type (WT)-p53 from the nucleus and eventually targets p53 protein for proteasomal degradation [1]. The anti-p53 functions of mot-2 in patients with colon cancer make mot-2 a potentially targetable oncoprotein for such patients [2, 3]. We have previously shown that a Ubiquitin-like (UBX)-domain-containing protein, UBXN2A, binds to and inactivates the mot-2 oncoprotein. UBXN2A expression is sufficient to re-activate WT-p53 through a mechanism of competitive interaction with mot-2 [4]. This novel protein complex (UBXN2A-mot-2) exists only in the cytoplasm of cancer cells and not in normal cells due to the dominant mitochondrial localization of mot-2 in normal cells [5]. Therefore, therapy that targets the UBXN2A-mot-2 complex will not affect normal cells. Previously, MKT-077 and withanone were reported as mot-2 small molecule inhibitors [6, 7]. However, several unacceptable side effects were observed when MKT-077 was used in a clinical trial [8].

In this study, we performed a high-throughput drug screen in order to look for a drug that targets UBXN2A. Veratridine (VTD), a plant alkaloid, was revealed to enhance transactivation of a UBXN2A promoter-luciferase reporter construct. Cell-based assays coupled with gene-specific shRNA silencing confirmed that VTD was sufficient to induce UBXN2A protein levels, resulting in colon cancer cell death in UBXN2A- and mot-2-dependent manners. Unlike cancer cells with a high level of mot-2 in the cytoplasm [2], non-cancerous cells remain intact when exposed to VTD. This study shows that VTD exhibits synergistic effects when combined with chemotherapeutic drugs. VTD potentiates the antitumor effect of 5-FU in long-term exposure and may affect CD44+ stem cells through the UBXN2A/mot-2-dependent pathway. The ability of VTD to induce UBXN2A expression, causing significant cancer cell death alongside standard chemotherapy, suggests VTD could be a potential complementary strategy in the treatment of solid tumors with high levels of mot-2 [9–12].

## RESULTS

### Elevated levels of mot-2 in clinical samples of colon and breast cancer are associated with cancer progression

**Figure 1:**
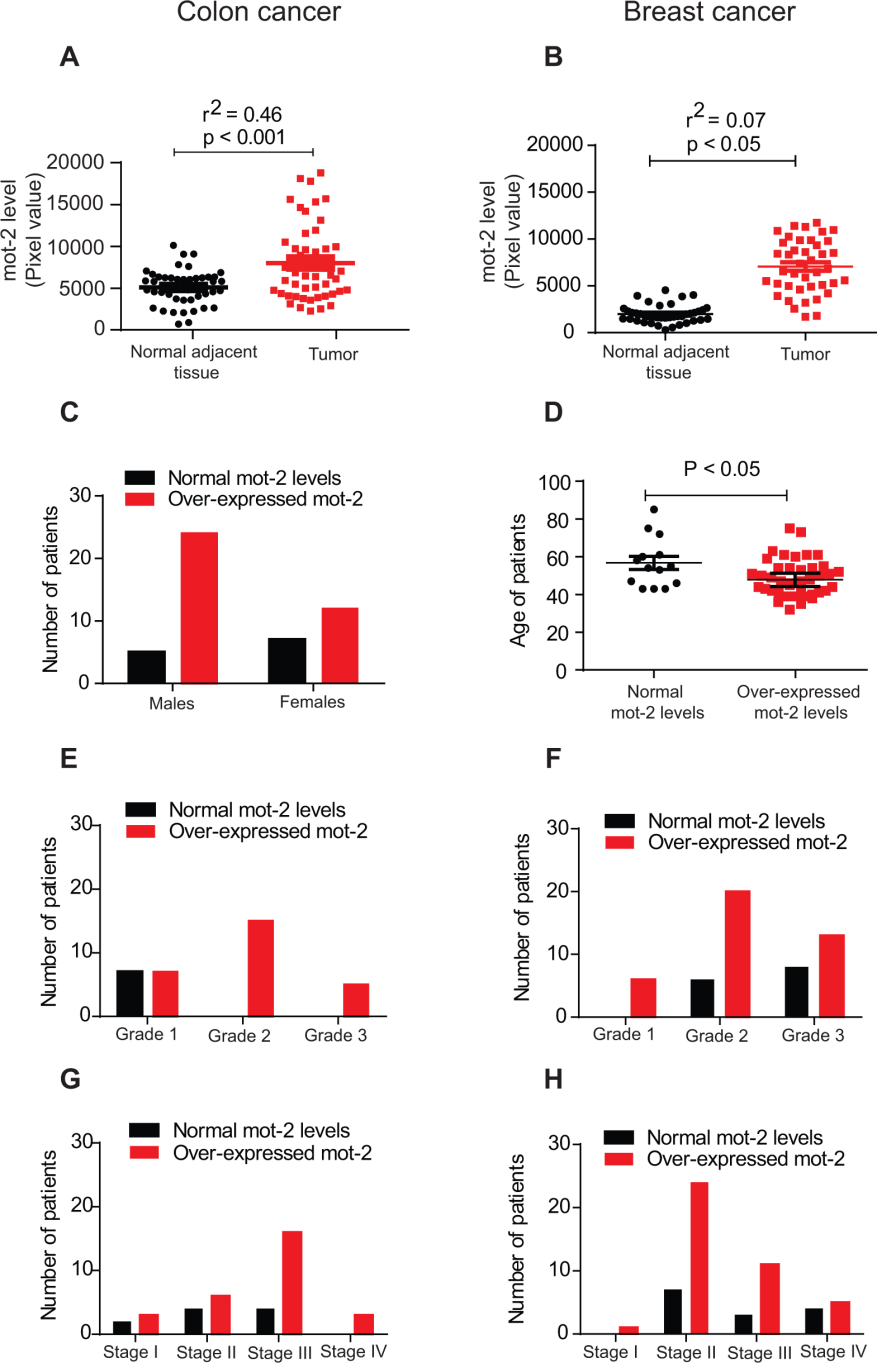
The heat shock protein mot-2 plays a critical role in tumorigenesis Equal amounts of tumor tissue lysates from 48 patients with colon cancer (A) and 55 patients with breast cancer (B) along with matched adjacent normal tissue lysates were probed with anti-mot-2 and anti-actin antibodies followed by quantitation and normalization of signals. **A–B.** These two diagrams show densitometry-quantified signals of mot-2 expression in tumor tissue versus normal adjacent tissue. The expression of mot-2 in the tumor tissues of patients diagnosed with colon cancer (A) and breast cancer (B) was significantly higher as compared to normal tissues (Colon tumor: R^2^ = 0.46 and *P* < 0.001, Breast tumor: R^2^ = 0.07 and *P* < 0.05, correlation coefficient test for each pair of variables). **C.** Overexpression of mot-2 in colon cancer is gender-dependent. Higher numbers of male patients show overexpression of mot-2 as compared to female patients. **D.** Overexpression of mot-2 in breast cancer is age-dependent. The statistical analysis shows that a relatively higher number of young female patients showed over-expression of mot-2 levels. **E–H.** Profiling of grade- and stage-dependent of tumors with colon (E and G) and breast (F and H) cancer showed that patients with mot-2 overexpression have been diagnosed with a higher grade and stage of tumors, indicating poorer survival. These clinical results confirm mot-2 as a potential target for the treatment of cancers and a promising prognostic factor.

We conducted a series of protein arrays (Supplementary Figure S1, Panels A–B) to compare the level of mot-2 in tumor tissue versus adjacent normal tissues. The results indicated that 75% (36 out of 48) of colon tumors show 1.5 fold or greater overexpression of mot-2 as compared to their normal adjacent tissues, with a maximum of 7.6 fold (*P* < 0.001, Figure 1A and Supplementary Table S1). We observed similar upregulation of mot-2 protein in breast cancer patients (*P* < 0.05, Figure 1B, and Supplementary Table S2). The increase in mot-2 levels was found to be higher among the tumor tissues of male patients with colon cancer as compared to female patients (Figure 1C). As described previously [13], the incidence and subsites of colon cancer are gender dependent due to several factors, including oral contraceptive use and hormone replacement therapy in females. The higher numbers of tumors with high levels of mot-2 proteins in male patients can be considered as an addition factor contributing to the reported differences between male and female [14]. Consequently, this can justify alternative treatment strategies based on the gender of colon cancer patients, as previously highlighted [15]. Further studies need to be done to explain whether the mot-2 level has any correlation with other observed differences such as the lower number of tumors in distal subsites observed in females versus males.

Moreover, upregulation of mot-2 only in breast cancer patients was age-dependent. The level of mot-2 was found to be significantly higher in comparatively younger females with an average age of 48.71 ± 1.54 years (*P* < 0.05, Figure 1D). The increased number of breast tumors with high mot-2 proteins in younger female patients that can be associated with poorer output might be related to a risk of specific tumor subtypes [16]. Breast tumors with upregulated mot-2 in younger patients can be a decent criteria for choosing different target therapies based on the age of patients, as some common markers are not age dependent in patients with breast cancer [17]. Further study needs to be conducted to explain the key mechanism underlying mot-2 alteration in younger patients with breast cancer. The upregulation of mot-2 in both colon and breast cancers (Figure 1E–1H), is grade- and stage-dependent indicating its prognostic value. These *in vivo* results indicate mot-2 as a significantly expressed oncoprotein in colon cancer with a potential to serve as a therapeutic target.

### Induction of the anti-mot-2 protein UBXN2A suppresses tumor growth in xenografts

**Figure 2:**
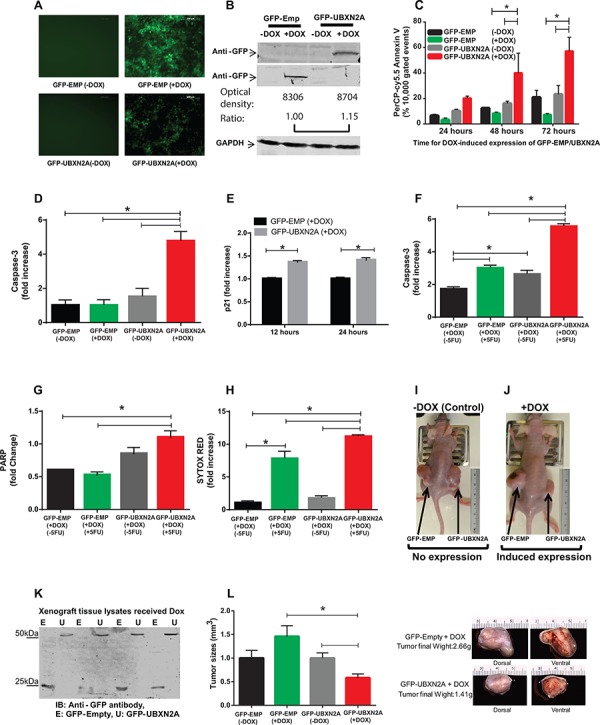
Induction of UBXN2A slows the growth of a colon cancer tumor ex vivo and in a mouse xenograft model by 50% **A.** A Tet-on regulated inducible HCT-116 colon cancer cell line was established successfully. 48 hours incubation with Doxycycline (DOX, 10 mg/ml) induces an equal expression of GFP-empty (EMP) or GFP-UBXN2A in HCT-116 cells. Images were taken using an inverted fluorescent microscope. **B.** WB showing the expression of GFP-empty and GFP-UBXN2A proteins in HCT-116 cells when treated with DOX for 48 hours. An anti-GFP antibody was used to detect the levels of GFP alone or of GFP-UBXN2A fusion proteins, while GAPDH was used as a loading control. Optical density of bands were measured by Image Studio software **C.** Inducible cells (GFP-EMP or GFP-UBXN2A) were treated with DOX for the indicated times. Cells were then stained with PerCP-Cy5.5 Annexin V antibody, and a total of 10, 000 gated events were analyzed by flow cytometry. DOX-induced UBXN2A expression in 48 and 72 hours showed a significant increase in annexin binding in those cells. The data is shown as mean ± SEM of three independent experiments in trplicate (*n* = 3) where *p < 0.001 using Tukey's modified student's t-test. **D.** Inducible cells (GFP-EMP or GFP-UBXN2A) were treated with DOX for 48 hours, followed by staining with Alexa Fluor 546 anti-caspase-3 antibody. Cells were analyzed using flow cytometry and collected data was normalized with GFP-EMP (+DOX). The DOX-induced UBXN2A expression significantly increased caspase-3 levels (*n* = 3, *p < 0.05). **E.** Inducible cells (GFP-EMP or GFP-UBXN2A) were treated with DOX for the indicated times. Immunofluoresence of p21 was performed. Intensity of fluorochrome signals from a confocal microscope were analyzed by the Image J (NIH, Bethesda, MD) program and plotted. The data was normalized with GFP-EMP (+DOX). The expression of UBXN2A significantly increased levels of p21 (*n* = 100, *p < 0.001). **F–G.** HCT-116 cells expressing GFP-EMP and GFP-UBXN2A under DOX were treated with 5-FU (100 μM) for 48 hours. Cells were then stained with Alexa Fluor 546 anti-caspase-3 (F) or anti-cleaved PARP (G) antibodies, followed by flow cytometry analysis. The induced UBXN2A significantly enhanced 5-FU-induced increase in caspase-3 and cleaved PARP levels (*n* = 3, *p < 0.05). **H.** Inducible HCT-116 cells were incubated with DOX and 5-FU for 48 hours, stained with Sytox Red, and then the population of dead cells was analyzed using flow cytometry. UBXN2A significantly enhanced 5-FU-induced cell death (*n* = 3, *p < 0.05). **I–J.** 1 × 107 Tet-on inducible cells were injected subcutaneously into the lower flanks of nude mice. The animals with palpable tumors (< 5 mm3) for early-stage tumor response were divided into two groups after injection to be fed with a standard diet (controls) or a DOX-containing diet (625 mg/kg). Tumor volumes in mm3 were determined with digital calipers by the formula Volume = (width) 2 × length/2 every second day for 40 days. **K.** Expression of GFP-empty or GFP-UBXN2A proteins in dissected xenografts confirmed successful tumor responses to DOX after 40 days. **L.** Growth of tumors with and without DOX on day 40. Treatment with DOX significantly decreases tumor size and weight by more than 50%. The data is shown as mean ± SEM of 9 mice (*n* = 4 for control and *n* = 5 for DOX) where *p < 0.05 using Tukey's modified student's *t*-test.

Several of the UBX-domain-containing proteins play positive or negative regulatory roles in diverse types of cancers [4, 18–20]. We generated two Tet-on inducible HCT-116 colon cell lines expressing GFP-empty or GFP-UBXN2A. Fluorescent microscopy and western blot (WB) analysis showed that incubation with Doxycycline (DOX) for 48 hours induces expression of GFP-empty or GFP-UBXN2A in HCT-116 cells (Figure 2A–2B). Quantitation of protein bands in Figure 2B showed there is no significant difference between GFP-empty and GFP-UBXN2A signals after doxycycline induction, which eliminates GFP involvement in the phenotypes presented in Figure 2. To measure the total level of p53 proteins in Tet-on inducible cells, we decided to use a flow cytometry approach as described by Brotherick et al [21]. This approach allowed us to measure p53 signals in cells gated for GFP expression; therefore, we obtained an accurate level of increased p53 expression in cells expressing GFP-UBXN2A versus GFP-empty. Supplementary Figure S1C shows significant upregulation of p53 expression by GFP-UBXN2A after induction with DOX.

Incubation of cells with DOX for 48 and 72 hours significantly increased early apoptosis in GFP-UBXN2A-expressing cells using Annexin V marker (Figure 2C and Supplementary Figure S2A–S2C). Flow-cytometry results further confirmed that expression of an apoptotic marker, caspase-3, significantly increased following DOX-induced UBXN2A expression (Figure 2D and Supplementary Figure S3A). Because our previous data [4] and others' [22] indicate reactivation of p53 upon mot-2 inhibition targets both cell proliferation and apoptosis, we further investigated whether DOX-induced UBXN2A expression affects cell cycle arrest in addition to the activation of the apoptosis pathway. Staining of the p21 cell cycle arrest marker showed that induction of UBXN2A expression increased expression of the p21 protein, which can lead to arresting cell growth (Figure 2E). Because the anti-cancer function of UBXN2A is mediated through mot-2-p53, we hypothesized that UBXN2A induction would potentiate the cytotoxic effects of standard chemotherapy which cause DNA damage and activate pathways that signal to p53 [2]. To answer this question, we examined apoptosis and cell death markers in UBXN2A-induced cells treated with 5-FU, a thymidylate synthase inhibitor commonly used in patients with colon cancer. Our flow cytometry data indicated that expression of capase-3 and PARP (apoptosis markers) and Sytox red (cell death marker) were significantly higher when UBXN2A-induced cells incubated with 5-FU (Figure 2F–2H).

We next examined how induction of UBXN2A contributes to tumor suppression in xenograft mouse models. After injection of inducible cells subcutaneously into the lower flanks of nude mice, mice with a palpable tumor volume (∼5 mm, early-staged tumor experiments [23]) were fed either normal or DOX diets for 40 days. The data confirmed that induction of UBXN2A can slow the growth of tumors (Figure 2I–2J). WB of dissected tumors confirmed successful induction of GFP-empty and GFP-UBXN2A (Figure 2K). Measurement of the growth rate of tumors showed that induction of UBXN2A led to a 50% reduction of tumor size and mass as well as the central necrosis in some mice 40 days after implantation (Figure 2L). In an advanced-staged tumor response approach [23], we confirmed that induction of UBXN2A can lead to more apoptotic/necrotic events during the development of stablished tumors using PSVue 794, a distinct marker of apoptosis/necrosis [24] used in live mice (Supplementary Figure S3B–S3F).

While the above results indicate that p53 has a role during UBXN2A activation, several evidence in the literature indicate inhibition of mot-2 can also lead to apoptotic events in the presence of mutant p53 protein [25]. To better understand the observed phenotype following expression of GFP-UBXN2A, we overexpressed UBXN2A by transient transfection of HCT-116 p53 −/− and p53 +/+ with GFP-Empty and GFP-UBXN2A cloned in pAcGFP1-C1 vector (Clontech). Forty-eight hours after transfection, cells were subjected to flow-cytometry analysis using Annexin V early apoptotic marker. Panels G and H in Supplementary Figure S3 showed expression of GFP-UBXN2A can induce early apoptosis in both p53 −/− and p53 +/+ HCT-116 cells. Induction of UBXN2A led to a two-fold increase in Annexin V positive cells in p53 (−/−) cells while UBXN2A showed a three-fold increase in Annexin V positive cells in p53 (+/+) cells. This later set of experiments (*n* = 3 in triplicate) confirmed the trend highlighted in our previous publication [4] and more importantly illustrates p53 is an important positive modulator in the UBXN2A-mot-2 pathway but that it is not an essential core component of UBXN2A's anti-cancer pathway.

### Discovery of UBXN2A enhancers using a luciferase-based assay

**Figure 3:**
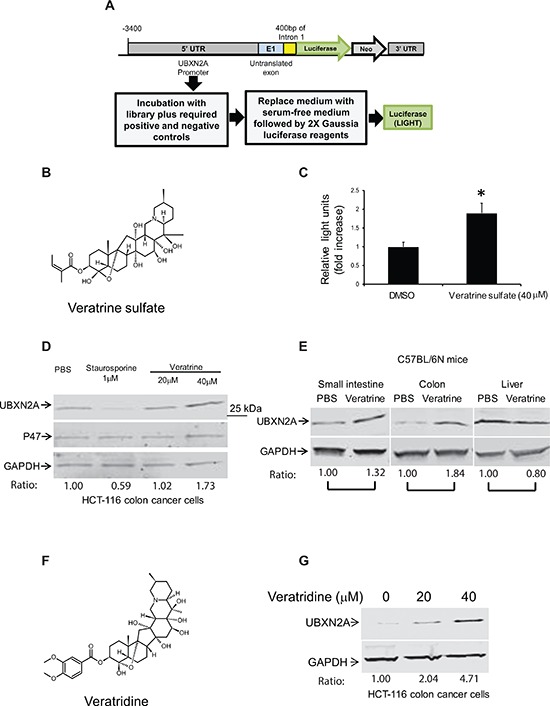
Veratrine and its purified form VTD increase UBXN2A level *in vitro* and *in vivo* **A.** A cell-based screen was conducted in search of compounds that induce the expression of the UBXN2A gene. The 3.9 K base of DNA upstream from the UBXN2A gene on human chromosome 2, including endogenous promoters and necessary enhancers as well as untranslated exon 1, was cloned into MCS-mGL.1, a Gaussia luciferase vector, and transiently transfected into HCT-116 colon cancer cells. We used empty MCS-mGL.1 for background expression. This cell line was used to screen over 1800 FDA (Food and Drug Administration) approved drugs, synthetic compounds, and natural products. A glow luciferase activity assay was conducted in triplicate. We found 12 potential candidates in the initial screen, which were confirmed again by the luciferase assay. **B–C.** 40 μM Veratrine sulfate (an unpurified form of VTD) resulted in a ∼twofold increase in luciferase activity when compared to control. **D.** WB experiments showed incubation of HCT-116 with Veratrine for 24 hours leads to up-regulation of UBXN2A, while Veratrine has no effect on p47 (UBXN2C), another member of UBXD family. GAPDH was used as a loading control. Staurosporine, as an alkaloid, was used as a negative control. **E.** IP injection of Veratrine (0.125 mg/kg) to C57Bl/6N mice for 28 days showed a selective upregulation of UBXN2A in small intestine and colon tissues, but no changes were observed in the liver of the same animals. **F.** VTD, a naturally occurring plant alkaloid of steroidal structure, is a component of Veratrine mixture. **G.** HCT-116 cells were treated with VTD (20 and 40 μM) and cell lysates were subjected to WB. GAPDH was used as a loading control. VTD increases UBXN2A protein levels in a dose-dependent manner.

Our *in vivo* results encouraged us to screen for compounds that can enhance expression of UBXN2A. Using the UBXN2A promoter upstream of a luciferase construct that was transiently transfected to HCT-116 cells, we performed a high-throughput drug screen with 1800 FDA approved drugs, synthetic compounds, and natural products. Forty-eight hours after transfection, cells were treated with compounds for 24 hours in triplicate (Figure 3A). The results showed that a 40 μM Veratrine sulfate (VH) resulted in a ∼two fold increase in luciferase activity (Figure 3B–3C). We looked at protein expression of UBXN2A as well as P47 (another member of the UBXD family) to confirm the selectivity of VH as a selective enhancer of UBXN2A protein in cells. The WB results showed that VH selectively upregulates UBXN2A (Figure 3D). Because Veratrine has a typical alkaloid structure and induces apoptosis, we looked for a similar structure with similar apoptotic outputs to use as a control alkaloid compound next to Veratrine in colon cancer cells. We chose staurosporine, since it is a microbial alkaloid compound and it induces apoptosis in colon cancer cells [26]. Staurosporine decreased UBXN2A level compared to cells incubated with Phosphate-buffered saline (PBS, Figure 3D). While these two compounds have a similar alkaloid base, they function differently in cells even in terms of genes involved in apoptosis [27]. It has been shown that staurosporine upregulates and downregulates several chaperone and endoplasmic reticulum proteins [28] during staurosporine-induced apoptosis. UBXN2A, as a co-chaperone of the endoplasmic reticulum associated degradation (ERAD) pathway [29], can be another target downregulated by staurosporine. Overall, these results indicate plant alkaloid Veratrine can selectively increase protein level of UBXN2A in HCT-116 colon cancer cells.

More importantly, intraperitoneal injection (IP) of 0.125 mg/kg VH [30] every day for 28 days leads to the selective upregulation of UBXN2A in small intestine and colon tissues of mice, while, due to a high pool of UBXN2A in the liver, these was no significant changes in the liver tissue (Figure 3E). We observed a different level of VH-dependent UBXN2A induction in different segments of the gastrointestinal system (esophagus to rectum; data not shown). These data suggest that VH can increase UBXN2A levels *in vivo* to a functionally meaningful degree, as we observed following UBXN2A induction in xenograft tissues (Figure 2). Of course, there are differences in terms of UBXN2A expression under the Tet-on inducible system, which uses human EF1 alpha promoter (lentiviral vector pLVi(3G), Clontech) versus the expression of endogenous UBXN2A by Veratrine, which activates UBXN2A's promoter.

The alkaloid fraction of Veratrine is known to contain a mixture of two major alkaloid esters: Veratridine (VTD) and Cevadine [31, 32]. VTD, with a veratroyl group at the 3-O-R1 position and a free hydroxyl group at the 15-O-R2 position can function as an antihypertensive plant-derived substance (Figure 3F) [33]. Besides antihypertensive functions, there is evidence for anti-proliferative and apoptotic roles of VTD in cancer cells [34–37]. The anti-proliferative and apoptotic functions of VTD are mediated by wild-type p53 [38]. Based on the above evidence, we hypothesized that the purified form of VH, VTD, is a major UBXN2A inducer. To answer this question, we treated HCT-116 cells with 20 and 40 μM of VTD for 24 hours. WB of the total cell lysate showed that VTD induces UBXN2A expression in a dose-dependent manner (Figure 3G).

Measurement of UBXN2A in cells and tissues (Figure 3) confirmed UBXN2A expression increases at least 1.84-fold in colon tissues (1.32-fold in small intestine and no changes in liver) in the presence of Veratrine (Figure 3E) and is increased 2–4 fold by its purified form, VTD, in HCT-116 colon cancer cells (Figure 3G). However, we observed lower UBXN2A expression with Veratrine (unpurified form of VTD) in HCT-116 cells (Figure 3D). This set of quantitation analysis (Figure 3D versus Figure 3G) further confirmed that VTD is the main UBXN2A enhancer in the Veratrine mixture, since VTD was more effective than Veratrine.

### VTD induces cell death of human colon cancer cells

**Figure 4:**
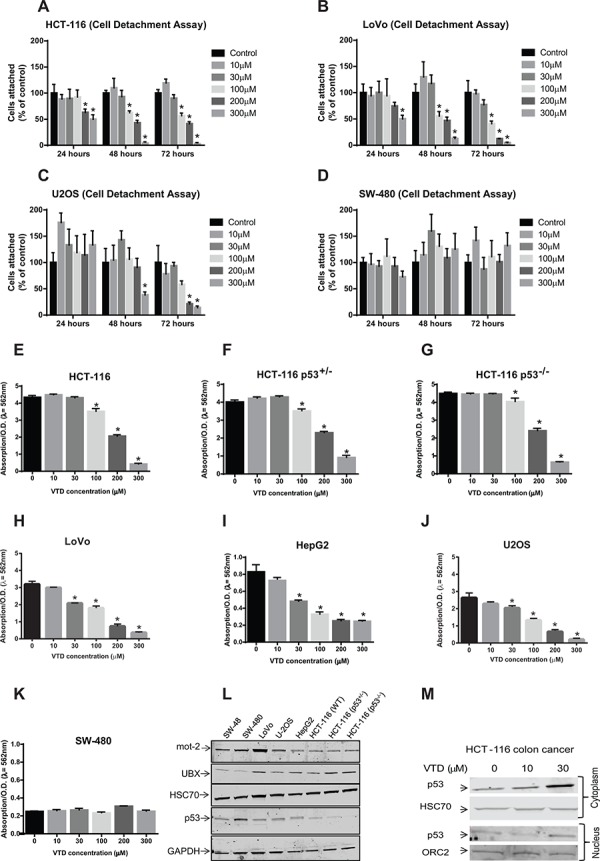
Induction of apoptosis and *in vitro* cytotoxicity by VTD in human cancer cell lines with different statuses of p53 and mot-2 **A–D.** Cells were treated with VTD for 24, 48, and 72 hours. After incubation, dead cells were washed off, and the remaining attached cells were calculated by counting in five random fields using the Axiovert 200 M inverted microscope and AxioVision software (cell detachment assay). The % of cells attached was calculated as % of control cells. VTD significantly decreases the % of cells attached in a dose-dependent manner in cancer cells with WT-p53 (**A**: HCT-116, **B**: LoVo and **C**: U2OS) but not with mutant p53 (**D**: SW480). The data in Panel D suggest Veratridine might promote cell proliferation. However, a strict statistical analysis rejects any significant changes in SW-480 cells. The data is shown as mean ± SEM of three independent experiments (*n* = 3, *p < 0.05). **E–K.** Cells were plated for 5 days, and the colonies of cells were treated with different concentration of VTD. The colonies of viable cells were stained with crystal violet dye and absorbance, as an index of measurement of colony forming units, was read at 562 nm (clonogenic survival assay). VTD induced a significant decrease in cell viability in a p53- and differentiation grade manner in HCT-116 poorly differentiated cells (E), HCT-116 p53^+/−^ (F), HCT-116 p53^−/−^ (G), LoVo well-differentiated cells (H), SW-480 (K), and two non-colon cancer cells: HepG2 (I) and U2OS (J) The data is shown as mean ± SEM of three independent experiments (*n* = 3, *p < 0.05). **L.** Determination of endogenous levels of mot-2, UBXN2A, HSC70, and p53 proteins in various cancer cell lines using WB. **M.** HCT-116 cells were treated with VTD (10 and 30 μM) and cytoplasmic and nuclear fractions were subjected to WB. HSC70 and Orc-2 antibodies were used as cytoplasmic and nuclear markers, respectively. VTD increases p53 in both cytoplasm and nucleus compartments in a dose-dependent manner.

Based on the above results, we then hypothesized that VTD-induced UBXN2A leads to apoptosis and cell death mimicking the Tet-on induced-UBXN2A model (Figure 2). As cell detachment and cell shrinking are ubiquitous aspects of apoptosis [39], we examined these features in four cancer cell lines: HCT116 (WT-p53), LoVo (WT-p53), SW-480 (mutant-p53), and U2OS (WT-p53), all in the presence of vehicle (DMSO, Supplementary Figure S1D), and different concentrations of VTD (Figure 4, Supplementary Figure S4). The results showed that VTD significantly increases cell detachment of HCT-116 (poorly-differentiated colon cancer cell line, Figure 4A), LoVo (well-differentiated colon cancer cell line, Figure 4B) and U2OS (osteosarcoma with perinuclear mot-2 expression [40, 41], Figure 4C) in a dose-dependent and time-dependent manner. In SW-480, VTD had no effect on cell detachment, indicating WT-p53 is one of the requirements for VTD's cytotoxic functions (Figure 4D). In addition, live cell imaging of cells incubated 72 hours with VTD revealed VTD induces cell shrinking and rounding resembling apoptosis changes in HCT-116 (Supplementary Figure S4A), LoVo (Supplementary Figure S4B), and U2OS (Supplementary Figure S4C) in a dose- and time-dependent manner, while SW-480 cells remain unchanged (Supplementary Figure S4D).

We next performed a clonogenic survival assay to determine colony formation in cancer cells in the presence and absence of VTD. Staurosporine-treated cells were used as a positive control (Supplementary Figure S4F). The results showed that VTD reduces colony formation in HCT-116 in higher doses. Using HCT-116 p53 +/− or p53 −/−, we showed that the anti-colony formation role of VTD is still partially p53 dependent in effective doses (Figure 4E–4G). Expectedly, VTD significantly decreases the number of colonies of LoVo (Figure 4H and Supplementary Figure S4E), HepG2 (liver cancer cell line, Figure 4I), and U2OS (Figure 4J) with as low as 30 μM VTD concentration, while SW480 cells with mutant p53 had no response to VTD, even at higher doses (Figure 4K). To further understand the level of p53's contribution in cell viability following Veratridine treatment, we treated two additional cell lines with different statuses of p53 (Supplementary Figure S5, panels A–B). As illustrated in the Supplementary Figure S5, the cytotoxic effect of VTD is cell dependent, since VTD successfully decreased colon formation in HT-29 colon cancer cells (Supplementary Figure S5, Panels A and C) carrying mutant p53. In opposite results, VTD had no significant cytotoxic effect on human pancreatic cancer cells (MIA PaCa-2, Supplementary Figure S5B) similar to SW-480 colon cancer cells (Figure 4K), both possessing a mutant p53 gene.

Despite a reasonable induction of UBXN2A by VTD in HCT-116 (Figure 3G), we did obtain a modest effect in HCT-116 poorly differentiated colon cancer cells (Figure 4A and 4E). In contrast, we observed a more distinct effect of VTD in LoVo well-differentiated colon cancer cells (Figure 4B and 4H). These results indicate that there are other conditions, such as the level of mot-2 in HCT-116 versus LoVo cells (Figure 4L) or the level of mot-2-p53 interaction [2], that can additionally modulate VTD cytotoxicity. In another word, the effect of VTD is cell-type dependent.

At the same time, we measured the mot-2, p53, and UBXN2A protein levels in all these cell lines. The WB analysis showed mot-2 levels vary based on the cancer cell type, while HSC70 is fairly equal in all examined cell lines (Figure 4L). To verify whether VTD-induced cell death is mediated through the UBXN2A-mot-2-p53 axis [4], we looked at the expression of p53 in the cytoplasm and nucleus of HCT-116 cells following VTD treatment. WB showed that VTD increases WT-p53 levels both in the cytoplasm and nucleus (Figure 4M) where p53 can activate its downstream cascades in dependent- or independent-transcriptional manners [42]. Due to a high cell death in higher doses of VTD, we could not use WB analysis for reliable p53 and UBXN2A detection and quantification. In addition, we looked at the p53 level in the absence of UBXN2A. LoVo colon cancer cells stably expressing shRNA against UBXN2A (clones 5 and 6) were treated with VTD (30 and 100 μM) followed by WB. Results presented in Supplementary Figure S4I indicate the expression of p53 is reduced in the absence of UBXN2A despite the presence of VTD.

Despite a reasonable upregulation of p53 in HCT-116 cells with 30 μM of VTD (Figure 4M) we observed VTD only induces cell death at higher does in HCT-116 cells (Figure 4E). It has been reported that VTD can induce cell death superoxide anions via induction of cytochrome c release in bovine chromaffin cells [37]. Therefore, it is possible that VTD uses a dual mechanism (mot-2-UBXN2A axis as well as superoxide anions) to promote cell death at higher doses in resistant cells such as HCT-116. Similar to a small-molecule Bcl-2 antagonist with duel pro-apoptotic functions in resistant cancer cells [43], the current study indicates that VTD can exhibit subcellular targeting effects that encompass two independent apoptotic pathways. As previously described, the level of mot-2-p53 binding under exogenous or endogenous stresses can also influence the p53-dependent apoptosis [2]. Therefore, despite increased levels of p53 in HCT-16 at lower doses following low doses of VTD (Figure 4M), it is possible that the status of the mot-2-p53 complex [2] or even the balances of other anti-apoptotic proteins [44] such as bcl-2 in resistant cells [45] sustain high p53 expression and its downstream functions. Furthermore, Supplementary Figure S6C–S6E shows a genotoxic agent, etoposide, is also more cytotoxic in well-differentiated cells (LoVo) and starts to kill LoVo cells at low concentrations (3 μM), while the same dose of etoposide was not effective in HCT-116 cells, similar to the effects observed with VTD alone.

### VTD is less cytotoxic towards non-cancerous cells and overexpression of mot-2 alleviates the cytotoxic effect of VTD

Because heat shock protein mot-2 is dominantly located in mitochondria in normal cells [5], we hypothesized that VTD-dependent upregulation of UBXN2A has no apparent sign of cytotoxicity in normal cells. To answer this question, we checked the effect of VTD on two non-cancerous cell lines. We incubated human umbilical vein endothelial cells (HUVECs), which mimic non-cancerous endothelial cells next to tumor cells, with VTD (S4G). The HUVEC cells did not respond to VTD in the first 24 hours, followed by a moderate response at the highest dose of VTD (300 μM) in 48 hours. We repeated the VTD experiments (0–300 μM) in non-cancerous kidney (HEK293) cells [46] (Supplementary Figure S4H), and we obtained similar results as those obtained in non-cancerous HUVEC cells (Supplementary Figure S4G). Both HUVEC and HEK-293 cells have been used extensively as control non-cancerous cells next to various cancer cell lines [47, 48]. Together, these results suggest VTD needs higher doses to trigger cell death in normal cells with low levels of cytoplasmic mot-2 and stresses as previously described [2]. In addition, these different levels of the stress condition [49] can justify increased sensitivity to VTD observed in LoVo and U2OS cells versus HCT-116 cells.

To further confirm mot-2 inhibition is a key event during VTD-induced cytotoxicity, in another set of experiments, we decided to conduct a gain of mot-2 functions in VTD treated cells. We first transiently transfected SW-48 well differentiated colon cancer cells with HA-empty or HA-mot-2 plasmids using Neon transfection system. Twenty-four hours after transfection, cells were treated with DMSO or VTD for another 48 hours. Cells were stained with Sytox red, followed by flow-cytometer analysis. Panel J in Supplementary Figure S4 shows that while VTD induces cell death in cells expressing HA-empty vector, the cytotoxic effect of VTD was significantly neutralized in cells expressing HA-mot-2 proteins. Expectedly, there were not significant differences in DMSO-treated cells expressing HA-empty or HA-mot-2 proteins.

### VTD induces apoptosis and cell death via the UBXN2A-mot-2-p53 axis

**Figure 5:**
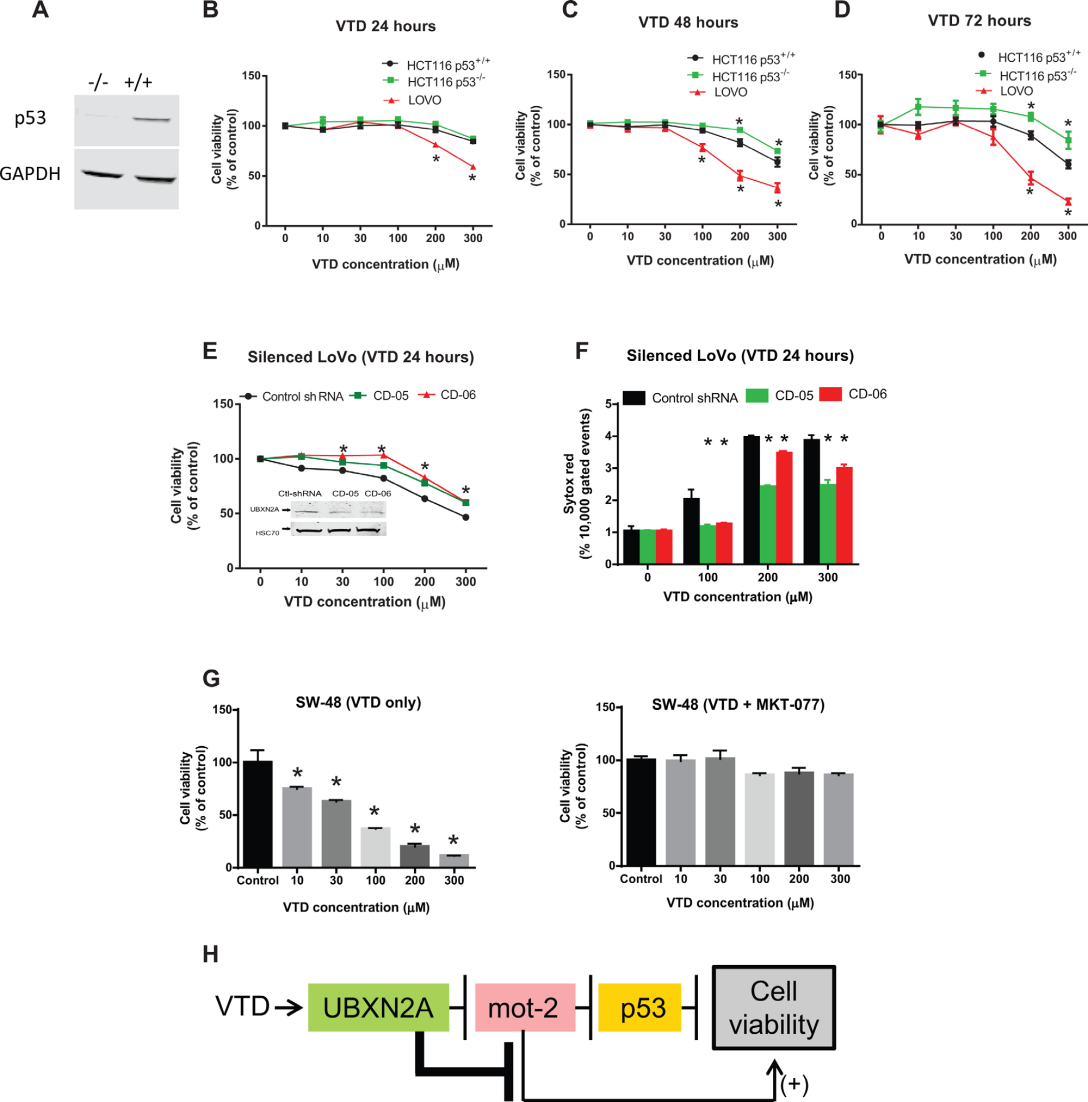
VTD functions via the UBXN2A-mot-2-p53 axis **A.** WB confirmed the presence and the absence of p53 protein in HCT-116 p53^(+/+)^ and HCT-116 p53^(−/−)^ cancer cell lines, respectively. **B–D.** The effect of VTD on the viability of poorly-differentiated (HCT-116 p53^(+/+)^ and HCT-116 p53 ^(−/−)^) and well-differentiated (LoVo) colon cancer cell lines was determined using an MTT assay. VTD's cytotoxic effect on all three cell lines was dose dependent in (B) 24 hours, (C) 48 hours, and (D) 72 hours. As compared to the HCT-116 p53^(+/+)^ cells, HCT-116 p53^(−/−)^ cells showed more resistance to VTD's effects, whereas well-differentiated (LoVo) colon cancer cells showed the highest sensitivity to VTD. **E.** LoVo cells were stably silenced for UBXN2A using a UBXN2A-shRNA along with a scrambled shRNA. Viability of UBXN2A-silenced cells was found to be significantly higher as compared to control cells upon treatment with VTD. **F.** In another set of experiments, UBXN2A-silenced cells were treated with VTD (100, 200, and 300 μM) for 24 hours. Cells were then labelled with Sytox Red followed by flow cytometry analysis. Results showed silencing of UBXN2A significantly decreases cell death in response to VTD. **G.** SW48 well-differentiated colon cancer cells with WT-p53 were treated with MKT-077 (5 μg/ml), a mot-2 inhibitor, along with VTD for 72 hours. A clonogenic survival assay showed VTD signifcantly decreases the colony number of SW-48. However, preincubation with MKT-077 neutralizes the cytotoxic effect of VTD. The data is shown as mean ± SEM of three independent experiments (*n* = 3) in triplicate where *p < 0.05 using Bonferroni's modified student's *t*-test. **H.** This flowchart recapped the sequence of proteins which activate and function upon VTD exposure to decrease cell viability.

We decided to confirm whether VTD-induced tumor suppression is indeed mediated via the UBXN2A-mot-2-p53 axis. After initial verification with WB (Figure 5A), HCT-116 (p53+/+ and p53 −/−) and LoVo were treated with VTD for 24, 48, and 72 hours (Figure 5B–5D). The MTT cell proliferation assay showed 24 hour treatment with VTD was not sufficient to produce any cytotoxic or anti-proliferative effect on HCT-116 cell lines; however, VTD at higher doses significantly decreases the viability of well-differentiated LoVo cells (Figure 5B–5D). All three cell lines started to respond to VTD at 48 hours, and reduction of cell viability reached a maximum at 72 hours, particularly in LoVo cells. We defined this phenomenon as a delayed cytotoxic response, since it occurs through UBXN2A transcription, as previously described for other anti-cancer agents [50]. The viability of HCT-116 p53^+/+^ was found to be significantly lower than HCT-116 p53^−/−^ in the presence of VTD, indicating that VTD partially acts via p53 in the cells. However, HCT-116 p53^−/−^ cells started to respond to VTD at higher doses after 48 hours, which indicates VTD can partly trigger cell cytotoxicity independent of the p53 mechanism (Figure 5D) probably due to UBXN2A-dependent inhibition of mot-2 unrelated to p53 sequestration (Figure 5H). In addition, these results indicate that the conditions of the well-differentiated colon cancer cells (LoVo) increases the effectiveness of VTD as described for other anti-cancer compounds [51]. In the second set of experiments, we treated two stable UBXN2A silenced clones of LoVo along with controls with VTD. We observed that UBXN2A-silenced cells showed greater cell viability than control cells, confirming that VTD requires UBXN2A to decrease the cell viability (Figure 5E). Besides the MTT assay, a set of Sytox red flow cytometry experiments further confirmed that in the absence of the UBXN2A there is a significant decrease in the cell death (Figure 5F; Supplementary Figure S5D–S5F). We observed that silencing of UBXN2A could not completely eliminate the cytotoxic effect of VTD in LoVo cells, particularly in higher doses. We believe that one reason for this issue is that our stable shRNA clones are not 100% silent for UBXN2A protein and therefore the remaining UBXN2A can still mediate the cytotoxic effect of VTD in a compensatory mechanism manner. It is noteworthy to indicate that we generated 10 UBXN2A silent cell lines with different levels of silenced UBXN2A. We examined the cytotoxic effect of VTD on all 10 cell lines that had different levels of UBXN2A. Interestingly, we found those silent cells with only 50% silenced UBXN2A or less were able to response to VTD, similar to the cells carrying scrambled shRNA.

To eliminate the possibility that VTD can inhibit mot-2 independently of UBXN2A, we decided to overexpress HA-empty or HA-mot-2 in LoVo stably expressing scrambled shRNA or shRNA against UBXN2A (Figure S6A). Among the available silent clones, we found Clone #6 (Figure 5E) is the ideal clone because UBXN2A shows maximum silencing. We transiently transfected cells with HA-empty and HA-mot-2 using the Neon transfection system (Life Technologies). Following initial optimization, we achieved high transfection efficiency (> 80%) and high cell viability (> 85%) simultaneously. After 24 hours cells were treated with DMSO or VTD for another 24 hours followed by flow-cytometry analysis using an Annexin V apoptosis marker. Panel A in Supplementary Figure S6 shows VTD (100 μM) successfully induced apoptosis in scrambled cell lines regardless of HA-empty or HA-mot-2 expression. However, in the absence of UBXN2A, VTD did not induce apoptosis, neither in cells expressing HA-empty nor in cells expressing HA-mot-2 proteins. Expectedly, there were also significant differences between cells expressing scrambled shRNA versus cells expressing UBXN2A shRNA regardless of the presence or the absence of exogenous mot-2. Together, this set of experiments further confirmed UBXN2A can play a critical role in anti-cancer mechanism of VTD in cancer cells.

Finally, to understand the contribution of mot-2 to the anti-cancer mechanism of VTD, we used a mot-2 inhibitor known as MKT-077 [7] along with VTD in well-differentiated SW-48 (WT-p53) colon cancer cells for 72 hours. Due to the critical role of mot-2 in the mitochondria, we could not use a silencing strategy for mot-2 in this set of experiments. The shRNA silencing of mot-2 interfered with normal mitochondrial functions and turned cells into an unhealthy and non-physiological status. VTD alone significantly decreased cell viability in a dose-dependent manner; however, upon mot-2 inhibition in the cytoplasm by MKT-077, VTD had no significant effect on cell viability (Figure 5G). It is noteworthy to specify that inhibition of mot-2 by MKT-077 indeed induced cell death in SW-48 cells by binding to mot-2 protein. However, adding VTD to MKT-077 did not increase any additional cell death. We concluded that pre-inhibition of mot-2 by MKT-077 hinders the effect of VTD, which needs mot-2 for its cytotoxic pathway. Together, these data show that VTD requires UBXN2A and mot-2 for its action while the presence of p53 can further enhance VTD's functions in a cell type-dependent manner (Figure 5H).

### Combination treatment with VTD and chemotherapeutic agents results in synergistic cytotoxicity in colon cancer cells

**Figure 6:**
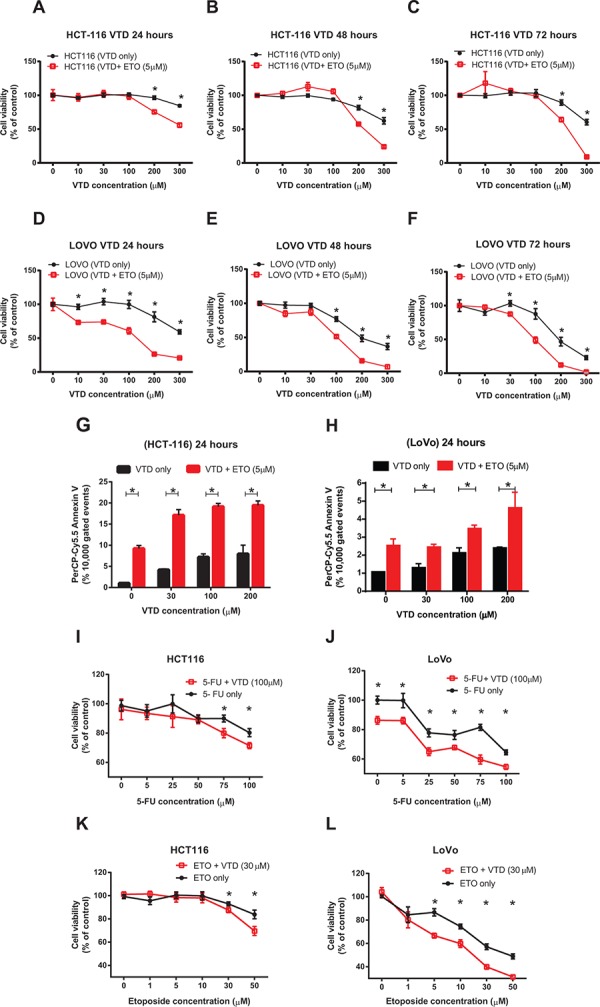
Synergistic inhibitory effects of VTD and etoposide or 5-FU chemotherapeutic drugs on the growth of human colon cancer cells **A–C.** HCT-116 and **D–F.** LoVo cells were treated with VTD (10- 300 μM) for 24, 48, and 72 hours. A suboptimal dose of Etoposide (ETO) (5 μM) was added to cells for the last 24 hours only. The viability of cells was determined as % of control (untreated cells) using an MTT assay. The chemotherapeutic drug ETO significantly enhanced the VTD-induced decrease in cell viability of both poorly differentiated (HCT-116) and well-differentiated (LoVo) colon cancer cell lines in a time-dependent manner. **G–H.** HCT-116 (G) and LoVo (H) cells were treated with VTD (10–200 μM) and a suboptimal dose of ETO (5 μM) for 24 hours. Cells were then stained with Annexin V apoptotic marker. ETO significantly sensitizes both poorly and well-differentiated colon cancer cells to VTD even at 30 μM. **I–L.** HCT-116 and LoVo cells were treated with different doses of 5-FU (5–100 μM, I-J) and ETO (1–50 μM, K-L) along with VTD (30 μM and 100 μM respectively) for 24 hours. MTT assays showed the treatment with 5-FU and ETO with VTD decreased cell viability at much lower drug doses particularly in LoVo cells. Results were analyzed using CalcuSyn software to calculate the combination index (CI) to confirm and quantify the synergism observed with combination therapies (S6 and S7 and Supplementary Tables 4 and 5). The data is shown as mean ± SEM of three different experiments (*n* = 3) where *p < 0.001 using Bonferroni's modified student's *t*-test.

Targeting carcinogenic-specific mechanisms by novel natural products, alone or in combination with standard chemotherapies, may provide synergy with existing treatments, lessen side effects, and ultimately improve both life expectancy and quality of life for cancer patients [52]. To examine whether VTD has synergism or additive effects with standard chemotherapeutic drugs commonly used in colon cancer patients, we first combined different doses of VTD with suboptimal doses of etoposide as determined by an MTT assay in HCT-116 versus LoVo cells in a time-dependent manner (Supplementary Figure S6C–S6E). Combination of VTD with a suboptimal dose of etoposide significantly potentiates the cytotoxic effect of VTD in HCT-116 (Figure 6A–6C) and LoVo (Figure 6D–6F). We analyzed our results with CalcuSyn software in order to understand the mode of interaction of the tested drugs (Supplementary Figure S6F–S6G). The median-effect plots for HCT-116 revealed that it was synergistic effect only when we mixed higher concentrations of VTD with sub-optimal concentrations of etoposide (5 μM) as the combination indexes (CI) were found to be ≤ 0.4 (Supplementary Figure S6F and Supplementary Table 4). However, in LoVo cells, lower concentration of VTD (100 μM) still showed synergistic effects (CI< 1) with 5 μM etoposide (Supplementary Figure S6G and Supplementary Table 4). The low doses of VTD produce an antagonistic effect (CI > 1) with etoposide (Supplementary Figure S6F–S6G), as previously described for other combination therapies [53]. Besides the cell viability assay, we decided to measure the apoptotic activity in the above combination therapy. The flow cytometry data revealed that combined treatment with VTD and a suboptimal dose of Etoposide (5 μM) for 24 hours significantly increased early apoptosis in HCT-116 (Figure 6G) cells as well as LoVo (Figure 6H).

While we observed a promising synergism effect with a combination of VTD and a suboptimal dose of etoposide, we decided to repeat the combination therapy in the presence of a low dose of VTD and clinical doses of 5-FU and etoposide. We hypothesized that the suboptimal dose of VTD, capable of increasing UBXN2A (Figure 3), would potentiate the cytotoxic effect of the chemotherapeutic drugs. Cells were treated with 5-FU (5–100 μM) and etoposide (1–50 μM) along with VTD (30 and 100 μM respectively) in two different sets of experiments for 24 hours. We found both the intermediate-dose of VTD (100 μM) and low dose (30 μM) of VTD significantly enhanced the 5-FU and etoposide effects on cell viability in both HCT-116 and LoVo cells (Figure 6I–6L and Supplementary Figure S6B).

We purposely showed the mild effect of VTD+5-FU/etoposide observed in HCT-116 poorly differentiated colon cancer cells (Figure 6I and 6K) next to LoVo well-differentiated colon cancer cells, which effectively responded to VTD+5-FU/etoposide (Figure 6J and 6L). The different sensitivity levels observed in HCT-116 and LoVo cells following VTD ± genotoxic agents further support our initial hypothesis that the effect of VTD can be cell dependent and determined by mot-2 [2] and other mot-2-related cellular pathways.

The analysis of our results with CalcuSyn software revealed that most of the combinations of 5-FU or etoposide with VTD have a synergistic effect on colon cancer cell viability (S7). In the case of HCT-116 cells, the strongest synergistic effect (CI = 0.165 and 0.109) was found when the highest concentrations of 5-FU (75 and 100 *μ*M) were combined with 100 *μ*M of VTD, while the synergistic effect started at lower combinations (Supplementary Figure S7A and Supplementary Table 5). When VTD (30 μM) was used along with etoposide, the combined effect was synergistic (CI = 0.516) at as low as 5 μM of etoposide (Supplementary Figure S7C and Supplementary Table 5). The same analysis for LoVo cells demonstrated that the combinations of even the smallest concentrations of 5-FU (5 *μ*M) with 100 μM VTD (CI = 0.32) and etoposide (1 μM) with 30 μM VTD (CI = 0.275) had a synergistic effect on the viability of well-differentiated colon cancer cells (Supplementary Figures S7B, S7D and Supplementary Table 5).

### VTD potentiates the cytotoxic effect of sub-optimal chemotherapy against cancer cells receiving long-term therapy

**Figure 7:**
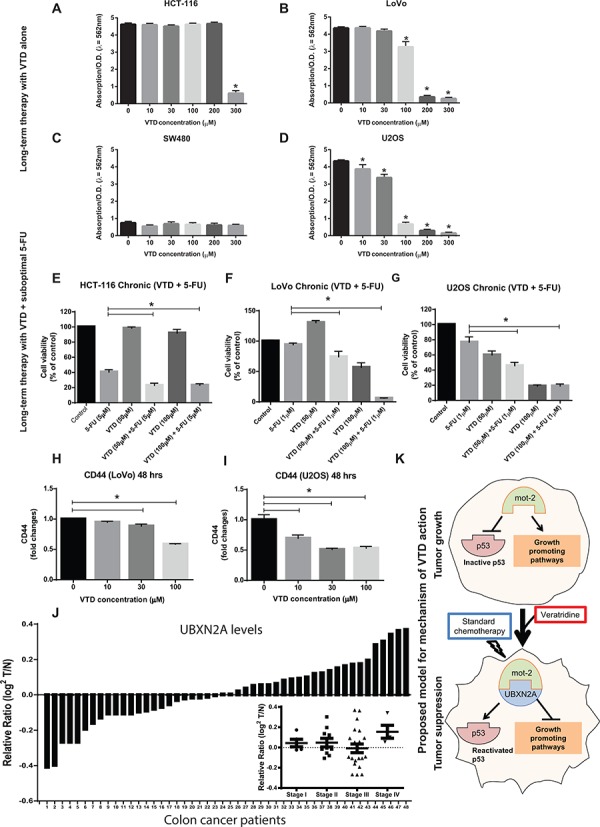
Sensitization of colon cancer cells to a long-term suboptimal dose of 5-fluorouracil exposure when combined with VTD **A–D.** (A) HCT-116, (B) LoVo, (C) SW-480, and (D) U2OS were treated with VTD (10–300 μM) for 10 days. Colony formation assays showed VTD effectively reduces cell viability in a cell type-dependent manner. **E–G.** (E) HCT-116, (F) LoVo, and (G) U-2OS were treated with suboptimal doses of 5-FU and VTD (50 and 100 μM) for 10 days. Clonogenic survival assays revealed an intermediate-dose of VTD significantly potentiates the standard chemotherapy used at very low dose (1–5 μM 5-FU). **H–I.** A series of flow cytometry assays using an antibody against the CD44 cancer stem cell marker illustrated that VTD can target CD44+ cancer stem cells and eventually eliminate them in a dose-dependent manner, implicating VTD as a potential cancer stem cell-targeting therapy. **J.** Tumor tissue lysates from 48 patients with colon cancer alongside the matched adjacent normal colon tissue lysates were probed with anti-UBXN2A and anti-Actin antibodies, followed by quantitation and normalization of signals. UBXN2A expression shows a marked downregulation in ≥ 50% of patients with colon cancer. UBXN2A expression levels may have a correlation with the stages of colon cancer as several patients at stage III had low level of UBXN2A (inset). (I) The proposed mechanism action of VTD. VTD increases the expression of UBXN2A, which releases the p53 from mot-2′s sequestration and, together with standard chemotherapy, can cause an effective tumor suppression.

Because colon cancer cells develop resistance to chemotherapy after an initial response [45, 54], we decided to investigate whether VTD can potentiate the cytotoxic effect of 5-FU when cells receiving long-term therapy mimicking the *in vivo* therapy. Cells were treated with VTD for 10 days. The cell viability assay showed that HCT-116 cells only responded to a high concentration of VTD, and they were able to recover at lower doses of VTD (Figure 7A). On the other hand, well-differentiated LoVo cells (Figure 7B) and an osteosarcoma U2OS cell line (with high perinuclear mot-2, Figure 7D) showed a significant decrease in cell viability in a dose-dependent manner with long-term exposure to VTD. Expectedly, the SW480 (mutant p53) showed no response to long-term exposure of VTD (Figure 7C).

Based on the significant effect of long-term exposure in LoVo and U2OS, we hypothesized that VTD could be a potential complementary strategy alongside 5-FU, an effective drug [55] with a high rate of resistant events [45] in colon tumors. To answer this question, we first determined the minimum concentration of 5-FU that has no effect or a very mild effect on cell viability after 10 days' exposure in three cell lines HCT-116, LoVo, and U2OS (data not shown). We performed 10 day treatments using a combination of a low dose of VTD (50 μM or 100 μM) plus a suboptimal dose of 5-FU (1 μM or 5 μM). The presence of VTD significantly potentiated the cytotoxic effect of the suboptimal dose of 5-FU (Figure 7E–7G). These results indicate that the novel anti-cancer mechanism of VTD can lower the dose of 5-FU in patients and thereby decrease side effects while postponing drug resistance. Because CD44+ stem cells are one of the major stem cells in cancers involved in self-renewal capacity, enhanced tumor initiation, and drug resistance [56], we decided to examine whether VTD can target these specific populations. Figure 7H shows that a high dose of VTD (100 μM) significantly reduces the abundance of the CD44+ cancer stem cells in LoVo cells. On the other hand, Figure 7I demonstrates that an intermediate-dose of VTD significantly reduces the abundance of the CD44+ cancer stem cells in the U2OS cancer cell line.

Analysis of the Oncomine database revealed that UBXN2A expression is downregulated in some human cancers, including in patients with colon adenocarcinoma [57]. Furthermore, WB of 48 human tumor and adjacent normal tissue lysates verified a marked downregulation of UBXN2A in ∼50% of patients with colon cancer (Figure 7J). We also showed that UBXN2A expression levels change based on stage of colon cancer (Figure 7J-inset). Interestingly, these data suggested that UBXN2A changes may have a correlation with the stages of colon cancer, as several patients at stage III had a low level of UBXN2A (inset). As it has been reported for other anti-cancer proteins such as SP1 [58], UBXN2A's up- and down-regulation can coordinate with the stage of cancers. Patients with high UBXN2A proteins may delay tumor progression and prolong overall survival, and patients with a low level of UBXN2A are more often found in the late stage of tumors (progressive tumor). Similar subpopulations have been described for the protein level of Chloride channel accessory 1 (CLCA1) [59] and topoisomerase-1 (Topo1) [60] in colon cancer patients, leading to different prognostic Outcomes. Further analysis employing a higher number of patients with colon cancer is needed to determine the relation of UBXN2A protein levels and patient survival in different stages.

This clinical data highlights the beneficiary effect of VTD as a UBXN2A enhancer in patients with low levels of UBXN2A, and it could be used to overcome resistance to chemotherapies in patients, particularly those with over-expressed mot-2 (Figure 7K).

## DISCUSSION

Here, we identify and establish UBXN2A as a colon tumor suppressor in both *in vitro* and *in vivo* models. We showed that UBXN2A enhancement leads to apoptosis at the cellular level and in live animals, resulting in tumor growth suppression. More importantly, we found induction of UBXN2A enhances the cytotoxic effects of 5-FU.

Despite its anti-cancer role, we observed that 50% of patients with colon cancer have underexpressed UBXN2A in their tumors, while at the same time 75% of these patients have an overexpression of mot-2. This clinical evidence plus the anti-growth function of UBXN2A in xenograft tumors encouraged us to look for UBXN2A enhancers. Using a high-throughput drug screen, we found VTD as a potential UBXN2A enhancer. VTD is a natural plant alkaloid found in Liliaceae plants, and it has prospective anticancer properties [34–37]. Natural alkaloids, as anticancer agents, have already served as a rich reservoir for drug discovery [61, 62]. As described for other anti-cancer alkaloids [52, 63], our results indicate that VTD can mediate transcriptional activity of the UBXN2A promoter, increasing the UBXN2A protein level *in vitro* and *in vivo* and resulting in upregulation of p53 protein in both the cytoplasm and the nucleus compartment, where p53 induces apoptosis and cell death [4]. The anti-cancer function of VTD is mediated through the UBXNA-mot-2 axis. In addition, the heterozygous p53+/− and homozygous p53 −/− HCT-116 cell lines show an intermediate cytotoxic effect in the presence of VTD. These latter results indicate: 1) VTD function is partially dependent on p53 since it can mediate apoptosis in the absence of p53 as well as the presence of mutant p53 proteins which may still be functional [25]. 2) VTD-dependent expression of UBXN2A and consequent binding of UBXN2A to mot-2 interferes with the other tumorigenic functions of mot-2 beyond p53 reactivation as described previously [2, 22, 64–68].

Quantitation of UBXN2A level showed there are differences among cell lines expressing exogenous or endogenous UBXN2A (Figure 2K), mouse tissues (Figure 3E), and human tumors (Figure 7J) which could be due to the different regulatory pathways unique in each system. Certainly, UBXN2A expression under the Tet-on inducible system, which uses human EF1 alpha promoter (lentiviral vector pLVi(3G), Clontech) versus the expression of endogenous UBXN2A by VTD, which activates UBXN2A's promoter generate different levels of protein expression. However, the comparison of Annexin V early apoptosis events induced by exogenous GFP-UBXN2A (Figure 2C) versus endogenous UBXN2A activated through VTD (Figure 6G) indicates a similar percent change in apoptosis, which verifies UBXN2A's contributions to the apoptosis process in both systems.

In this study, we found that VTD-dependent inactivation of mot-2 has a synergetic effect with etoposide and 5-FU, two chemotherapeutic drugs with different anti-cancer mechanisms. Combination therapy of VTD and 5-FU or etoposide at clinical dosages as well as suboptimal doses confirmed VTD enhances the cytotoxicity of these two genotoxic agents. Significant reduction of cell viability with long-term exposure of suboptimal doses of 5-FU in the presence of low doses of VTD verified the clear synergistic effects of the two treatments combined. As previously rationalized [2], the UBXN2A-mot-2 dependent anti-cancer mechanism of VTD combined with DNA damage mechanisms triggered by conventional chemotherapy can be considered a novel treatment strategy wherein two different but interconnected pathways can selectively choose cancer cells with high levels of mot-2 and high pools of inactivated p53 versus normal cells with low mot-2 in the cytoplasm.

Recent chemotherapeutic studies confirmed traditional chemotherapies are not capable of eradicating cancer stem cells (CSCs) and fail to prevent disease relapse and metastatic dissemination, indicating that new therapies need to focus on the ability to target CSCs [69]. Mot-2 protein plays a protective role in cancer stem cells [70]. Depletion of CD44 positive stem cancer cells in the presence of VTD suggests this alkaloid may target a sub-population of cancer stem cells through the UBXN2A-mot-2 pathway as described for other natural products [71]. Further studies of both molecular and self-renewal [72] assays are needed to understand the specific inhibitory role of VTD in the progression of cancer stem cells.

In summary, successful tumor growth suppression of xenografts in the presence of induced UBXN2A led to a drug screen to identify a natural compound capable of upregulating UBXN2A protein in both *in vitro* and *in vivo* models. We found that VTD induces apoptosis and reduces cell viability in cancer cells and CSCs in a UBXN2A-, mot-2, and partially p53-dependent manner, while normal cells dominantly remain intact. Combination therapy of VTD and standard chemotherapy showed VTD or its modified analogs can be a complementary strategy alongside suboptimal dose of chemotherapy, particularly in well-differentiated colon tumors. This study establishes the concept that the anti-cancer protein UBXN2A plays a crucial opposite role in colon tumorigenesis, and it justifies the transition of a novel plant alkaloid compound to clinical development.

## MATERIALS AND METHODS

### Mot-2 and UBXN2A detection by protein microarray

The expression of mot-2, UBXN2A, and actin (for normalization) in normal and tumor tissues were determined according to the manufacturer's instructions (Protein Biotechnologies, CA, USA). The complete list of patients and their tumors are provided in Supplementary Table S1 and S2.

### Cell culture, chemicals, and drug treatments

Cells were purchased from American Type Culture Collection (VA, USA) and they were grown in the recommended medium. Veratridine, Veratrine, etoposide, 5-Fluorouracil and staurosporine were purchased from Sigma (MO, USA). Because we used DMSO as a solvent for VTD at different concentration (10–300 μM), we examined different concentrations of DMSO (0.02%–0.3%) corresponding to VTD used (10–300 μM) in HCT-116 cells. As the results show in Panel D of Supplementary Figure S1, concentrations of DMSO as a vehicle had no cytotoxic effect on HCT-116 colon cancer measured by MTT assay at 24, 48, and 72 hours. This set of data further confirmed the cytotoxic effect observed with VTD is purely related to the anti-cancer function of VTD and not DMSO.

### Antibodies and immunoblotting analysis

Nuclear and cytoplasmic fractions were prepared from the cells using NE-PER Nuclear and Cytoplasmic Extraction Reagents (Pierce, IL, USA) according to the manufacturer's instructions. Protein concentrations were determined by BCA assay followed by SDS-PAGE and WB analysis with the appropriate antibodies (Supplementary Table 3). Signals were acquired using Odyssey infrared imaging (LI-COR) and Image Studio software (version 3.1).

### High-throughput drug screening

A cell-based screen was conducted in search of compounds that induce the expression of the UBXN2A gene. The 3.9K base of DNA upstream from the UBXN2A gene on human chromosome 2, including endogenous promoters and necessary enhancers as well as untranslated exon 1 (Figure 3A), was cloned into MCS-mGL.1, a Gaussia luciferase vector, and transiently transfected into HCT-116 colon cancer cells, with empty MCS-mGL.1 for background expression. This cell line was used to screen over 1800 FDA (Food and Drug Administration) approved drugs, synthetic compounds, and natural products (Xactagen). A glow luciferase activity assay was conducted in triplicate followed by semi-quantitative RT-PCR and WB analysis for UBXN2A, P47 (negative control), or GAPDH± Veratrine or its purified form VTD.

### MTT cell viability assay

An MTT (3-[4, 5-dimethylthiazol-2- yl]-2, 5-diphenyltetrazolium bromide) assay was performed to measure the viability of cells. Briefly, cells were seeded at a density of 10 × 10^3^ cells per well in phenol-red free growth media. After 24 hours, cells were treated with various concentrations of VTD ± 5-FU or etoposide. After incubation, an MTT dye was added to the cells for 2–4 hours. The absorbance of samples was measured at 630 nm using an EXL808 absorption spectrophotometer (Biotek, Winooski). The viability of cells was calculated as % of control. Cell detachment and clonogenic survival assays were conducted as previously described [4]. Early apoptosis, caspase-3, cleaved PARP and Sytox Red (Life Technology, NY, USA) were measured by the Accuri C6 flow cytometer system (BD Pharmingen, MD, USA) [73].

### Xenograft models in nude mice

1 × 10^7^ Tet-on HCT-116 cells expressing GFP-empty or GFP-UBXN2A were injected into 6 to 8 week old nude female mice [athymic nude-Foxn1nu, Harlan (IN, USA)] by subcutaneous injection. The animals with palpable tumors (∼5 mm^3^) were then divided into two groups and fed with a standard diet (controls, *n* = 5) or a Dox-containing diet (625 mg/kg, *n* = 5). Of the control, one mouse was removed from the experiment due to a tumor size of > 200 mm^3^ at 25 days, and we had a total of 9 mice at day 40 with 18 tumors (one GFP-empty and one GFP UBXN2A per mouse). Tumor volumes were determined as previously described [4].

### Statistics

Statistical analysis was perfomed using GraphPad Prism 6.0 (GraphPad Prism Software, Inc.) where appropriate. Results are shown as mean ± SEM and are representative of at least three independent experiments with a P value less than 0.05. Calculated r2 is a measure of goodness-of-fit of linear regression.

## SUPPLEMENTARY FIGURES AND TABLES


