# Supplementary material for: A plant alkaloid, veratridine, potentiates cancer chemosensitivity by UBXN2A-dependent inhibition of an oncoprotein, mortalin-2
Source: Oncotarget. 2015 Jul 11;6(27):23561–81. doi: 10.18632/oncotarget.4452 (PMC4695137; doi:10.18632/oncotarget.4452)
Supplement: Supplementary file 1 [file oncotarget-06-23561-s001.pdf]

## SUPPLEMENTARY FIGURES AND TABLES

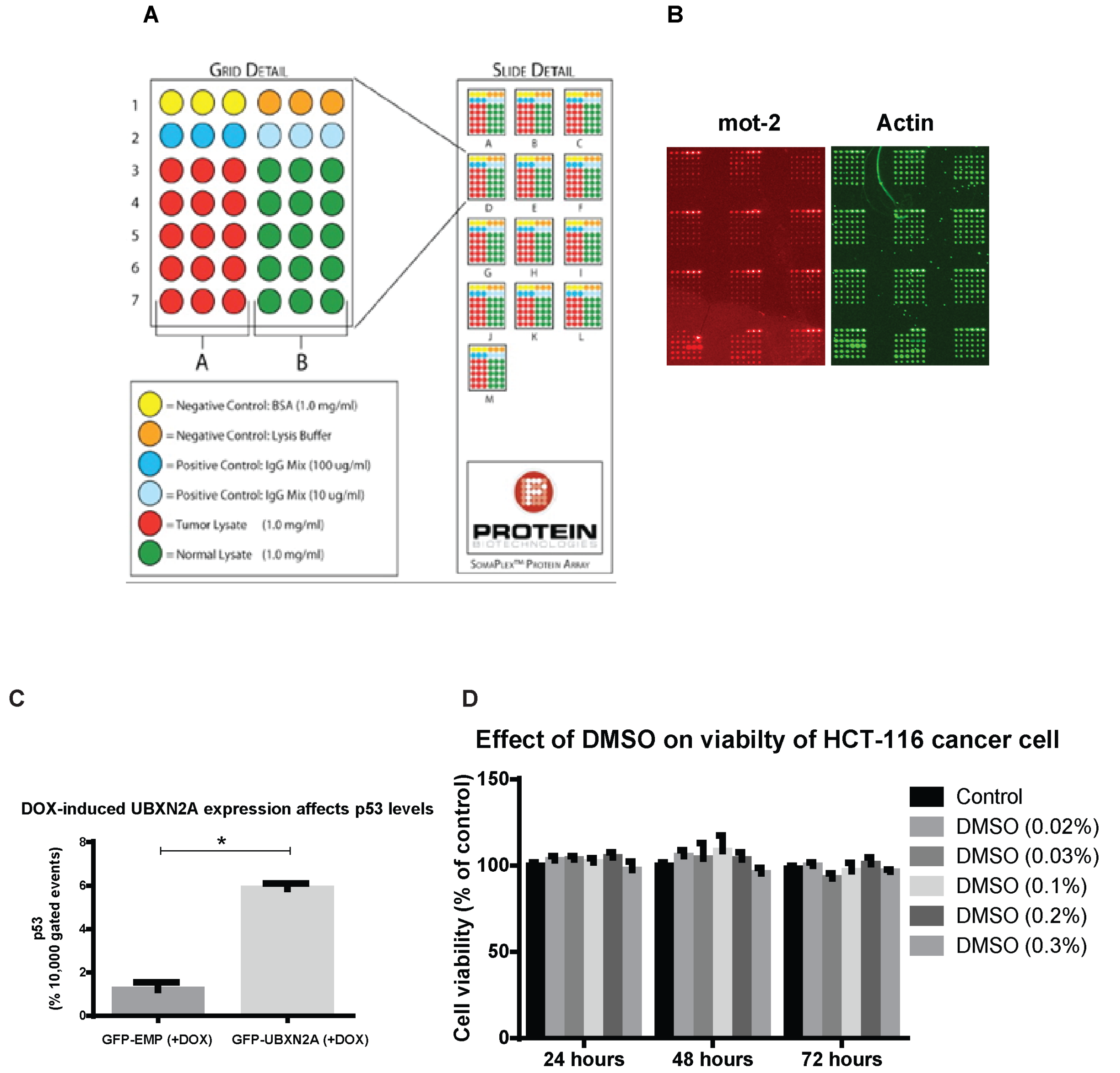


**Supplementary Figure S1: Protein-microarray experiment for the determination of mot-2 levels. A.** Schematic diagram of standard microarray slide. BSA and lysis buffer were used as negative controls (yellow spots), whereas a mixture of IgG was used as a positive control (blue spots). Tumor lysates (red spots) and normal lysates (green spots on the slide) are derived from high quality clinical specimens (Supplementary Tables S1 and S2). Each lysate is spotted at a single concentration (1.0 mg/ml) in RIPA buffer that permits most soluble proteins to retain their native, or non-denatured, structure and activity in many cases. Expression of mot-2 and UBXN2A were determined using a mouse anti-mot-2 and rabbit anti-UBXN2A antibodies, respectively. Visualization of antibody binding was accomplished using a LI-COR goat-anti-mouse or goat-anti-rabbit antibodies, and pixel numbers for each fluorescent band were measured with an automated Image Studio Ver3.1 digitizing system. After normalization with actin, a "fold-difference" in protein measurements was calculated by dividing the pixel numbers of the tumor sample to its matched normal sample. **B.** These two panels show the actual membranes with lysates of tumor and normal tissues probed by anti-mot-2 and anti-actin antibodies. Three individual membranes with tissue lysates from the same patients (48 colon cancer and 55 breast cancer) were analyzed for mot-2, UBXN2A, and actin levels as a loading control. **C.** To measure the total level of p53 proteins in Tet-on inducible cells, we used a flow cytometry approach as described by Brotherick et al [1]. p53 signals were measured in cells gated for GFP expression. Results showed significant upregulation of p53 expression by GFP-UBXN2A after induction with doxycycline. **D.** We used MTT assay to examine the cytotoxicity of different concentrations of DMSO (0.02%–0.3%) corresponding to VTD used (10~300 μM) in HCT-116 cells. Results confirmed concentrations of DMSO used as a vehicle had no cytotoxic effect on HCT-116 colon cancer measured at 24, 48, and 72 hours.

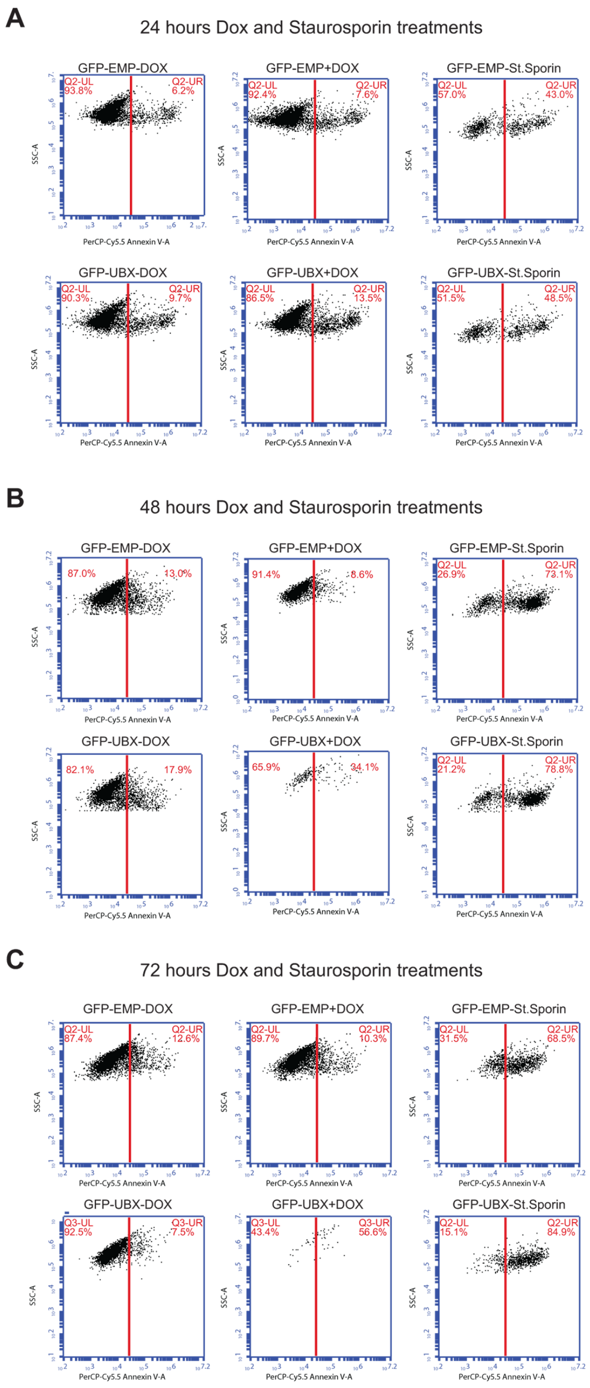


**Supplementary Figure S2: Induction of UBXN2A enhances annexin binding in HCT-116 cells.** Inducible HCT-116 cells (GFP-Empty (EMP) or GFP-UBXN2A) were treated with DOX for **A.** 24 hours, **B.** 48 hours, and **C.** 72 hours. Cells treated with media (left-side plots) and staurosporine (right-side plots) were used as negative and positive controls, respectively. Cells were then stained with PerCP-Cy5.5 Annexin V antibody and a total of 10, 000 gated events were analyzed by flow cytometry. DOX-induced expression of UBXN2A significantly increased the binding of annexin in a time-dependent manner. While we followed 1 μg/ml Doxycycline concentration as recommended by Clontech Laboratories, Inc., it has been reported that DOX can be toxic at concentrations of 1 μg/ml or even lower [2]. Therefore, this fact can explain a part of the lower recorded events in the presence of DOX in Figure 2C and Figure 2-supplement (Panels B and C). In addition, we observed under inverted fluorescent microscope that super high expression of GFP-empty or GFP-UBXN2A as an exogenous protein causes those cells to look unhealthy and small (exhausted cells). Due to the small size, these cells were not gated in our flow-cytometry, resulting in fewer recorded events in Panels B and C in Figure 2-supplement. However, despite a lesser count in the presence of DOX, we were able to have a minimum of 10,000 events during flow-cytometry analysis, which ultimately showed more apoptotic events in the presence of GFP-UBXN2A in Figure 2C.

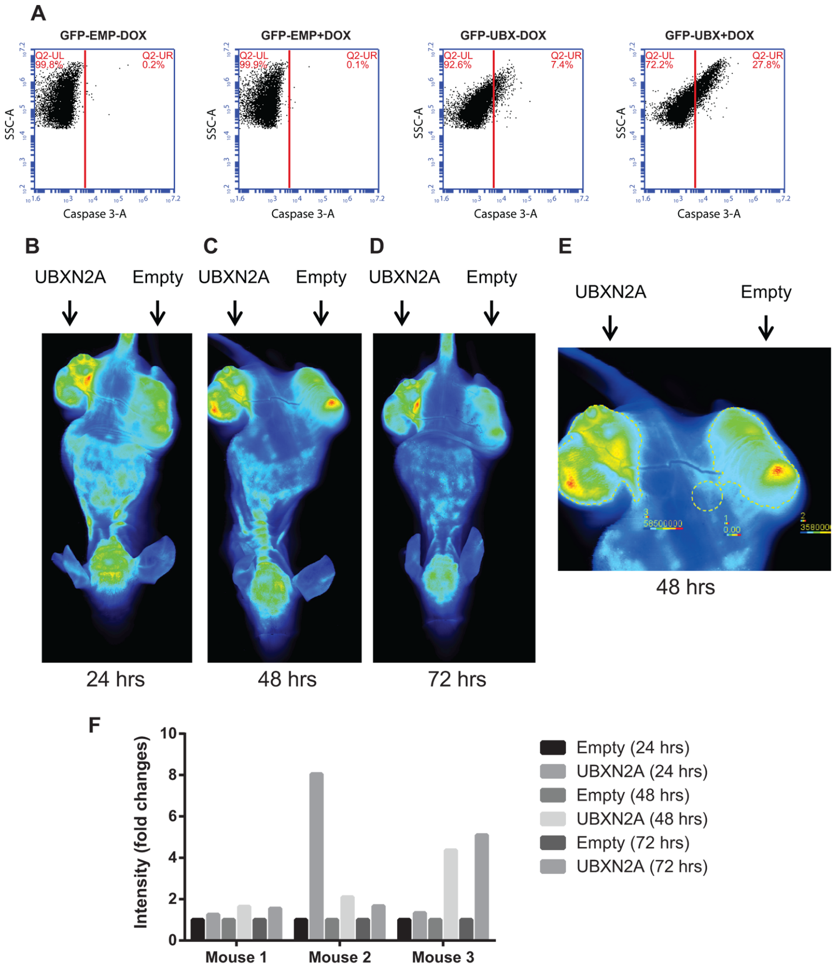


**Supplementary Figure S3: Induction of UBXN2A enhances apoptosis in *in vivo* and *ex vivo* models of colon cancer. A.** HCT-116 cells transfected with GFP-Empty (GFP-EMP) and GFP-UBXN2A (GFP-UBX) were treated with DOX for the indicated times. Cells were then stained with anti-caspase-3 and Alexa Flour 546 secondary antibodies. A total of 10, 000 gated events were analyzed. The treatment of HCT-116 cells transfected with GFP-UBXN2A DOX for 48 hours and 72 hours (data not shown) showed an increase in caspase-3 staining. **B–E.** Three athymic nude mice were injected subcutaneously with Tet-on inducible HCT-116 cells. The right flank received GFP-UBXN2A and the left flank received GFP-empty. We allowed tumors to reach 100 $mm^3$ in size (advanced-staged tumor experiments [3]) before feeding the mice with doxycycline food for 30 days. At day 30, mice were given 1 mM solution of PSVue 794 fluorescent probe intravenously via the tail vein. PSVue 794 [4], a distinct marker of apoptosis/necrosis, was used in live animals. Images were taken 24, 48, and 72 hours after injection of the agent. We followed the protocol provided by LI-COR (http://www.licor.com/bio/products/reagents/psvue_794/) to monitor apoptosis after UBXN2A induction. PSVue 794 is visible in the 800 channel of ODYSSEY CLx instruments. The 800 channel image display provides a green signal, which represents fluorescence from the PSVue 794. The Odyssey/Image Studio software allows the user to manipulate the green signal by pseudo color without changing the quantification values as describe in the software's instruction. Those manipulations can include changing the colors used (in this case, green and pseudo color) as well as changing the brightness and contrast of the image. Therefore, the quantified signal intensity values of the final image will be the same regardless of what display settings are in use. Also, the color bar shown within Panel E refers specifically to the fluorescence intensity of the PSVue 794 detected in each scanned image. Panel B-E represents signals collected in mouse 2. **F.** Relative fluorescence was calculated by dividing the signal from the UBXN2A-containing tumor with signals from the tumor containing GFP-empty vector, as described by the manufacturer (LI-COR, inc). The relative fluorescence data shown for three individual mice indicated significant apoptosis/necrosis activity in tumors expressing GFP-UBXN2A versus GFP-empty. We had different staining patterns in these three individual mice, which may be due to the vascularity of tumors as well as general circulation which can be different in each examined mouse, particularly in mice carrying a large tumor (> 100 $mm^3$). It has been shown that PS794 is applicable to *in vivo* imaging and targets both apoptotic and necrotic cells [5]. After tumor dissection, we also observed necrotic tissue characterized as dark liquefied tissue at the center of some well-stabilized xenografts [6], which could be another reason for different dissimilar levels of intensity between individual mice. This piece of *in vivo* data basically further confirms the *in vitro* and *in vivo* results presented in Figure 2, where we showed induction of UBXN2A induces apoptosis and consequently cell death). (*Continued*)

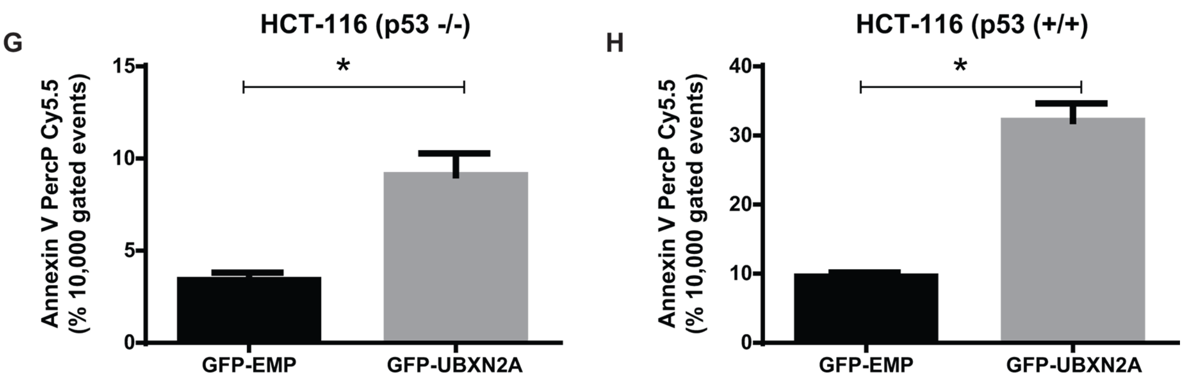


**Supplementary Figure S3: (*Continued*) Induction of UBXN2A enhances apoptosis in *in vivo* and *ex vivo* models of colon cancer. G–H.** HCT-116 p53 −/− and p53 +/+ cells were transiently transfected with GFP-Empty and GFP-UBXN2A cloned in pAcGFP1-C1 vector (Clontech). Forty-eight hours after transfection, cells were subjected to flow-cytometry analysis using Annexin V early apoptotic marker. Results showed expression of GFP-UBXN2A can induce early apoptosis in both p53 +/+ and p53 −/− HCT-116 cells (*n* = 3 in triplicate).

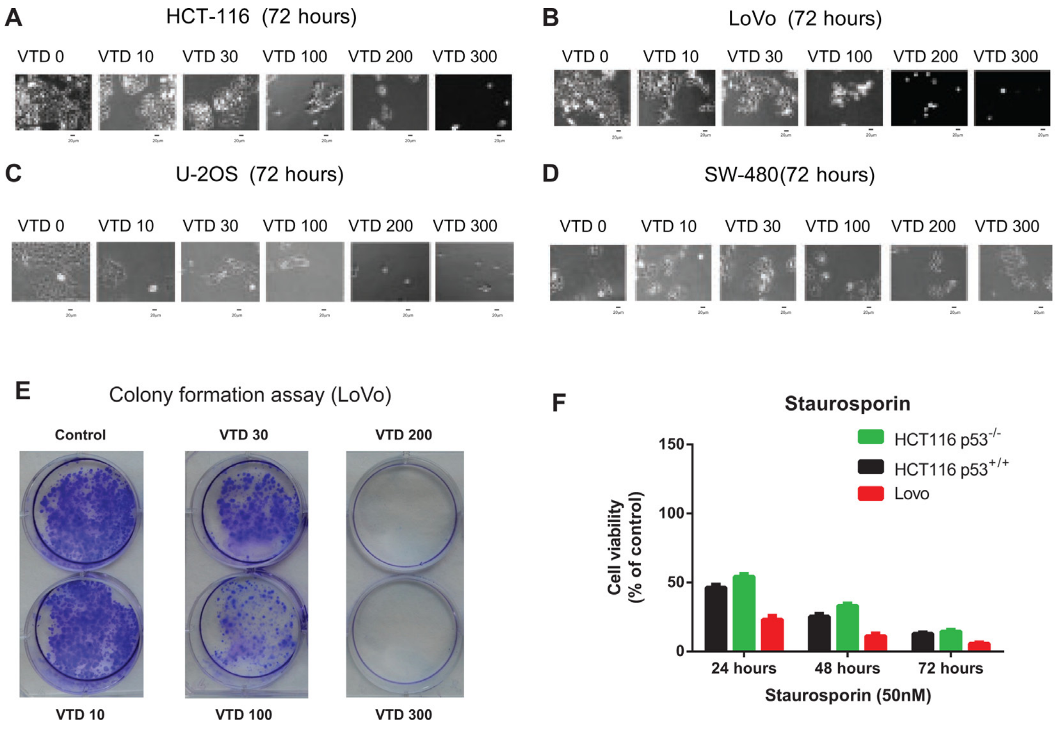


**Supplementary Figure S4: VTD causes morphological alterations of apoptotic cell death in cancer cells while normal cells remain intact. A.** HCT-116, **B.** LoVo, **C.** U2OS, and **D.** SW480 Cells were treated with VTD (10–300 μM) for 72 hours. Dead cells were washed off and remaining cells were observed under the light microscope. The treatment of HCT-116, LoVo, and U2OS cells with VTD increased the number of cells washed off (increased cell death) and hence decreased the number of cells attached to the surface. The remaining VTD-treated cells either appeared round or showed shrinkage, two common signs of apoptosis. The effect of VTD was time and dose dependent (24 and 48 hours data not shown). U2OS was shown to be the most affected cancer cell line by VTD among all. VTD did not have any effect on SW480, and these cells continued to grow independent of time and concentration of VTD. **E.** Representative images of a colony formation assay. LoVo cells were treated with VTD (10–300 μM) and a colony formation assay was performed as described in main Figure 4. **F.** Effect of staurosporine (50 nM), a known anti-cancer natural alkaloid (positive control) on the viability of poor-differentiated (HCT-116 $p53^{(+/+)}$ and HCT-116 $p53^{(-/-)}$) and well-differentiated (LoVo) colon cancer cell lines was determined by MTT assay. Cells were treated for 24, 48, and 72 hours. Cell viability was determined as % of control (untreated cells). Staurosporine significantly decreased the % cell viability of both HCT-116 and LoVo colon cancer cell lines in a time-dependent manner. The data is shown as mean ± SEM of three different experiments (*n* = 3). (*Continued*)

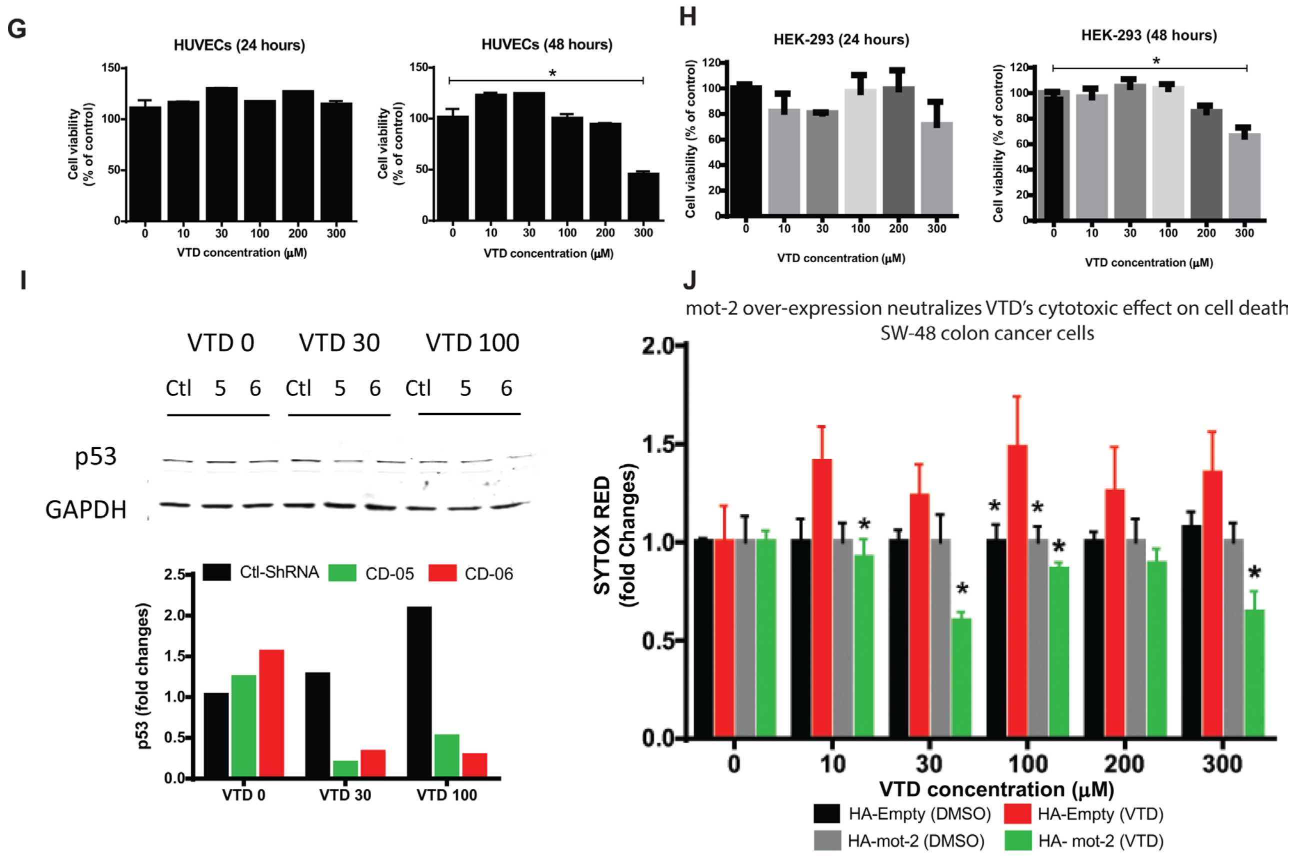


**Supplementary Figure S4: (*Continued*) VTD causes morphological alterations of apoptotic cell death in cancer cells while normal cells remain intact. G.** Because human umbilical vein endotheilal cells (HUVECs) have been used as a ideal transmigration model across endotelial cells and intestinal epitelum cells [7], we decided to investigate the effect of VTD on these relevant non-cancerous human cell lines. HUVECs cells were treated with various concentrations of VTD for 24 (left) and 48 hours (right). The viability of cells was determined by MTT assay, as described previously. VTD did not cause any changes to HUVEC cells' viability except for a very high concentration of VTD in 48 hours, which caused a significant decrease in cell viability. **H.** HEK-209 cells as another non-cancerous cell line were treated with different concentrations of VTD followed by MTT assay. Similar to HUVEC cells, HEK-293 cells' viability was affected for a very high concentration of VTD in 48 hours. The data (G and H) is shown as mean ± SEM of three different experiments ($n = 3$). **I.** LoVo cells stably expressing scambled shRNA (Ctl) or shRNA againt UBXN2A (clone 5 and 6) were treated with VTD for 24 hours followed by WB analysis. Results indicate the expression of p53 is reduced in the absence of UBXN2A. This experiment has been performed two times, and similar results have been obtained. We noticed the level of p53 slightly increased in the absence of VTD in silent cells. We have no scientific explanation for this p53 elevation in the absence of VTD. Further research is necessary to understand the functions of UBXN2A protein in cancer cells in the absence of exogenous stress. As part of the ubiquitin-proteasome pathway, UBXN2A might regulate the basal p53 protein level in unstressed cells independent of its role mediated through mot-2 inhibition. **J.** To examine whether overexpression of mot-2 protein can neutralize the VTD cytotoxic pathway, we first transiently transfected SW-48 cells with HA-empty or HA-mot-2 plasmids using Neon transfection system (Life Technologies). Following initial optimization, we achieved high transfection efficiency (> 80%) and high cell viability (> 85%) simultaneously. Twenty-four hours after transfection, cells were treated with DMSO or different concentration of VTD for another 48 hours. Cells were stained with Sytox red, followed by flow-cytometer analysis. Results show that while VTD induces cell death in cells expressing HA-empty vector, the cytotoxic effect of VTD was significantly neutralized in cells expressing HA-mot-2 proteins. There were not significant differences in DMSO-treated cells expressing HA-empty or HA-mot-2 proteins ($n = 3$).

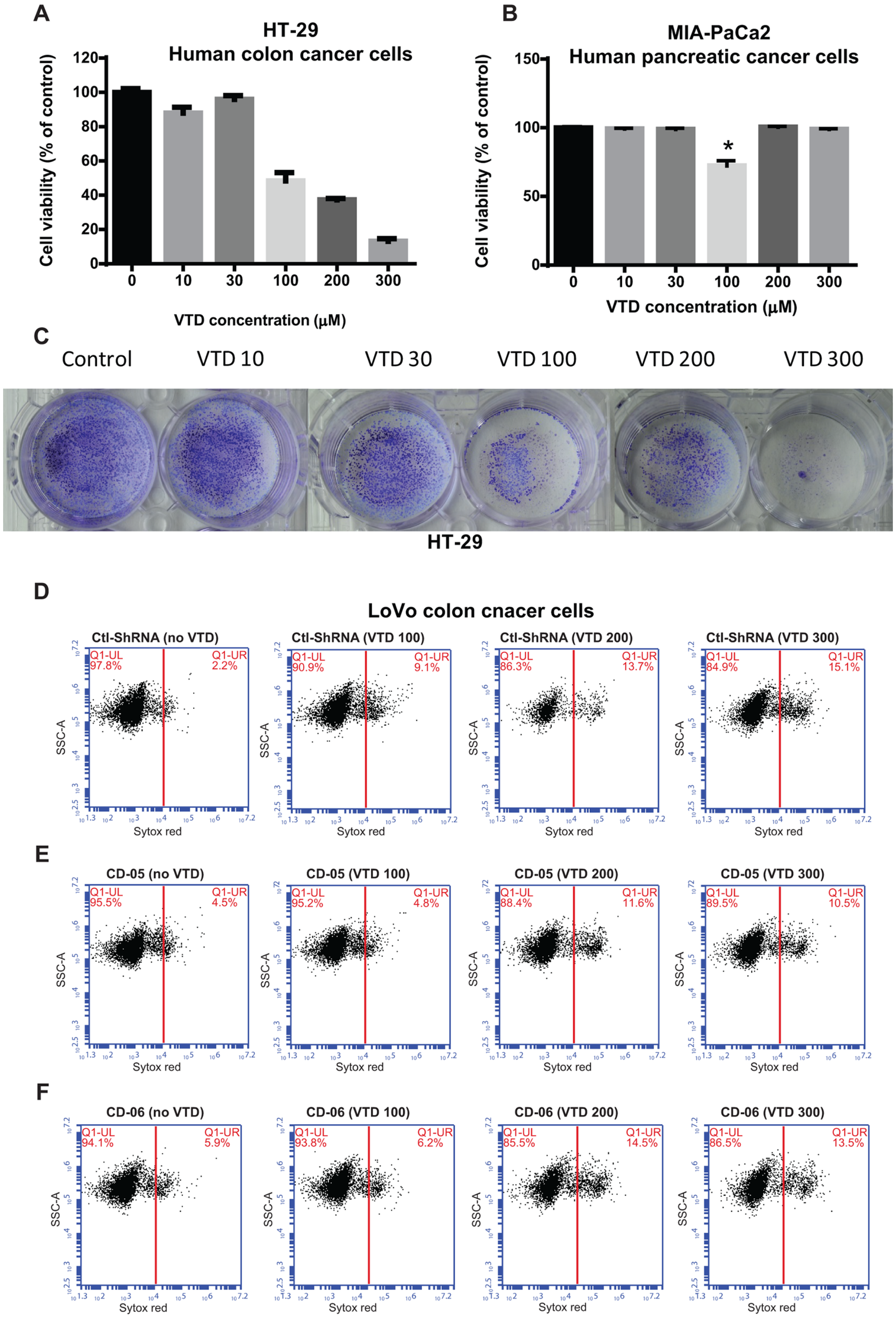


**Supplementary Figure S5: VTD induces cell death via the UBXN2A pathway. A–C.** We treated HT-29 colon cancer cell and MIA PaCa-2 human pancreatic cancer cells both possessing a mutant p53 gene. VTD successfully decreased colon formation in HT-29 colon cancer cells (Panels A and C). In opposite results, VTD had no significant cytotoxic effect on MIA PaCa-2 (Panel B). **D–F.** LoVo colon cancer cells were stably silenced for the UBXN2A gene. Cells with control (Ctl) ShRNA (D) and two UBXN2A-silenced clones, i.e., CD-05 clone (E) and CD-06 clone (F), were treated with VTD (100, 200, and 300 μM) for 24 hours. Cells were then labelled with Sytox Red for flow cytometry analysis. Cells positive for Sytox Red staining are shown on the right side of dot plots. The results showed that UBXN2A-silenced clones have lower Sytox Red staining as compared to control shRNA when treated with VTD.

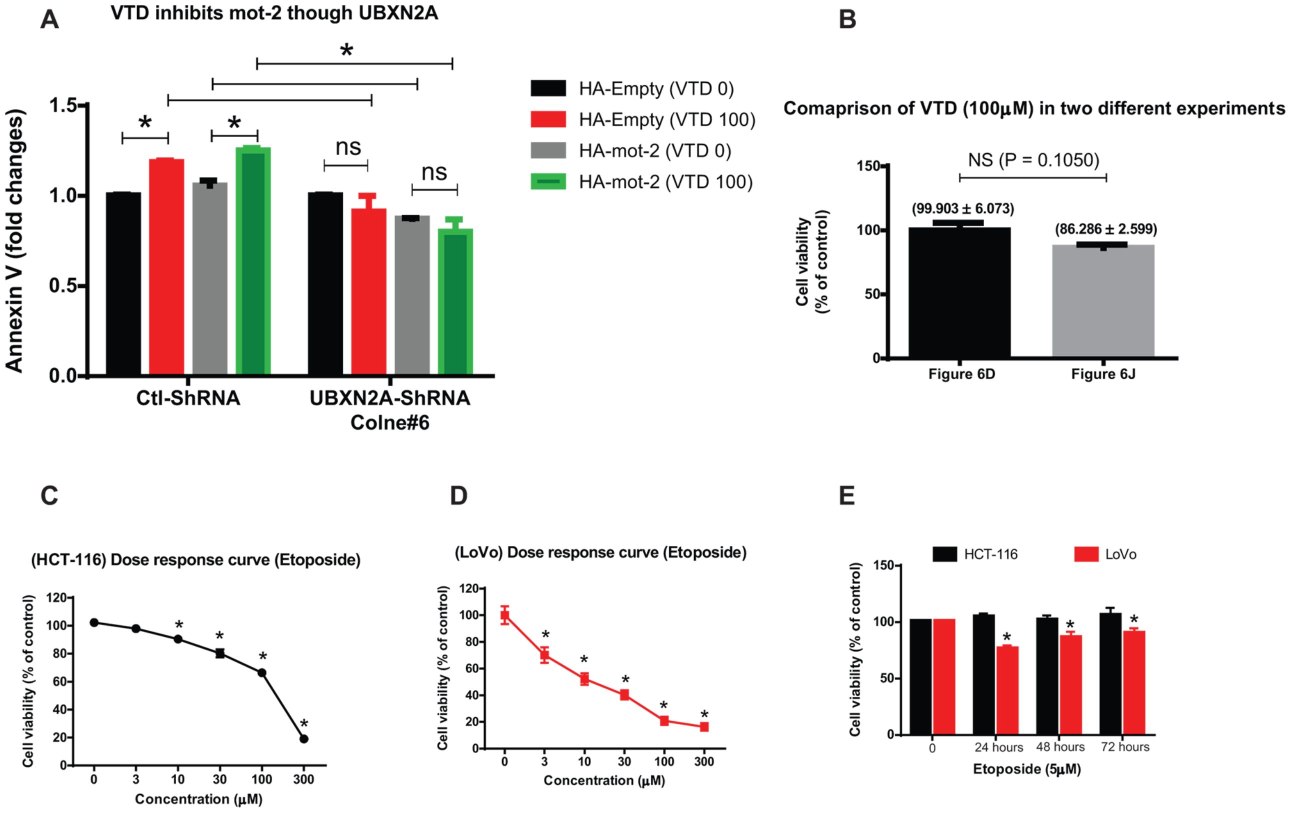


**Supplementary Figure S6: A sub-optimal dose of etoposide (ETO) enhances VTD's cytotoxic effect on colon cancer cells. A.** To eliminate the possibility that VTD can inhibit mot-2 independently of UBXN2A, we overexpressed HA-empty or HA-mot-2 in LoVo stably expressing scrambled shRNA or shRNA against UBXN2A (Clone #6) using the Neon transfection system. Clone #6 (Figure 5E) was the ideal clone because UBXN2A shows maximum silencing. After 24 hours cells were treated with DMSO or VTD for another 24 hours followed by flow-cytometry analysis using an Annexin V apoptosis marker. Results show VTD (100 µM) successfully induced apoptosis in control cell lines. However, in the absence of UBXN2A, VTD did not induce apoptosis, neither in cells expression HA-empty nor in cells expressing HA-mot-2 proteins. Expectedly, there were also significant differences between cells expressing scrambled shRNA versus cells expressing UBXN2A shRNA regardless of the presence or the absence of exogenous mot-2. Together, this set of experiments further confirmed UBXN2A can play a critical role in anti-cancer mechanism of VTD in cancer cells. **B.** We found LoVo cells (Figure 6J-main text) showed ~15% (86.286± 2.599) depletion in cell viability in response to 100 µM VTD. Similar LoVo cells in Figure 6D-main text showed lesser sensitivity to VTD (99.903 ± 6.073). To find whether this difference is significant, we compared these two individual data from two independent experiments and we observed no significant difference. This non-significant difference (~5% to 13.6%) could be due to the different passage numbers, the initial number of cells seeded onto 10-cm plates, and/or the density of cells exactly before VTD treatment. The overall messages provided by Figures 6D and 6J will stay the same: "VTD and standard chemotherapies (etoposide and 5-FU) significantly decrease cell viability in a synergetic manner." **C–D.** Dose response curves of ETO against poorly (HCT-116) and well-differentiated (LoVo) colon cancer cells. HCT-116 and LoVo cells were treated with ETO (3–300 µM) for 24 hours. The plots show that a concentration of ETO of ≥ 10 µM is sufficient to decrease the viability of HCT-116 cells (C), whereas a concentration of ETO as low as 3 µM is significantly effective on LoVo cells (D). **E.** Based on the response curve obtained in panels C and D, HCT-116 and LoVo cells were treated with a sub-optimal concentration of ETO for the indicated times. The treatment significantly decreased the viability of LoVo cells; however, it had no effect on HCT-116 cells. (*Continued*)

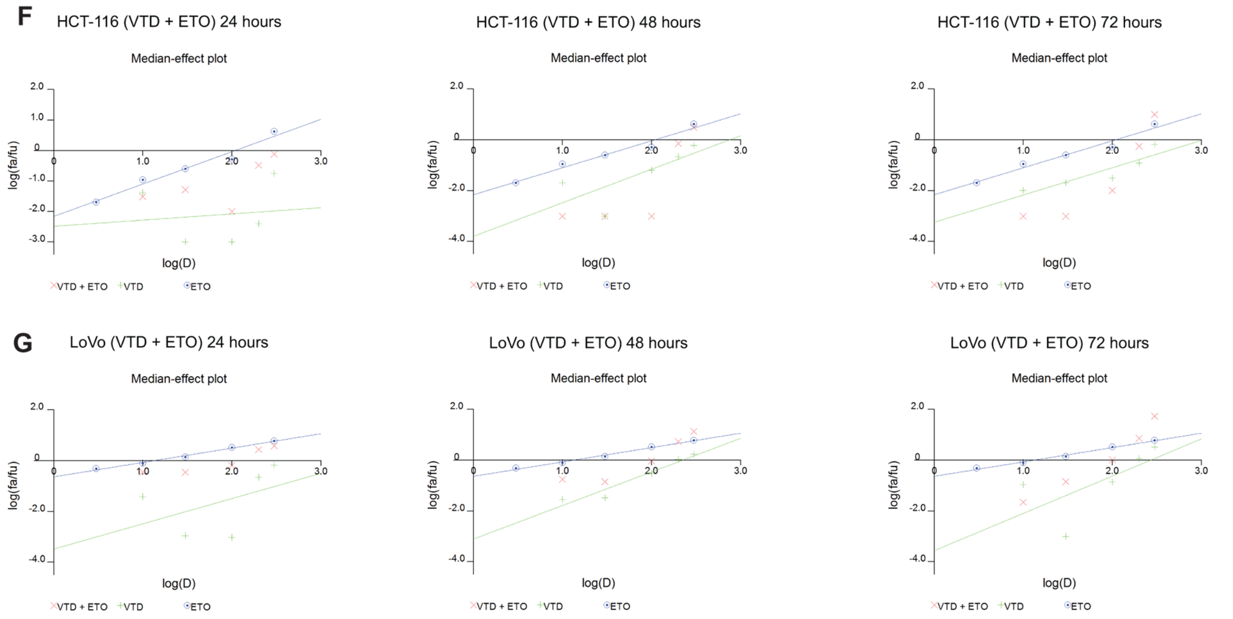


**Supplementary Figure S6: (*Continued*) A sub-optimal dose of etoposide (ETO) enhances VTD's cytotoxic effect on colon cancer cells. F–G.** Median-effect plots calculated by CalcuSyn software [8] determined the synergistic, additive, or antagonistic response of VTD and ETO on (F) HCT-116 and (G) LoVo when used in combination at 24, 48, and 72 hours. According to the CalcuSyn software, a combined treatment is synergistic when CI < 1. The average for three value (cell death) were applied as the experimental data to calculate median effect plot curves for each treatment as instructed by provided instruction available in the software.

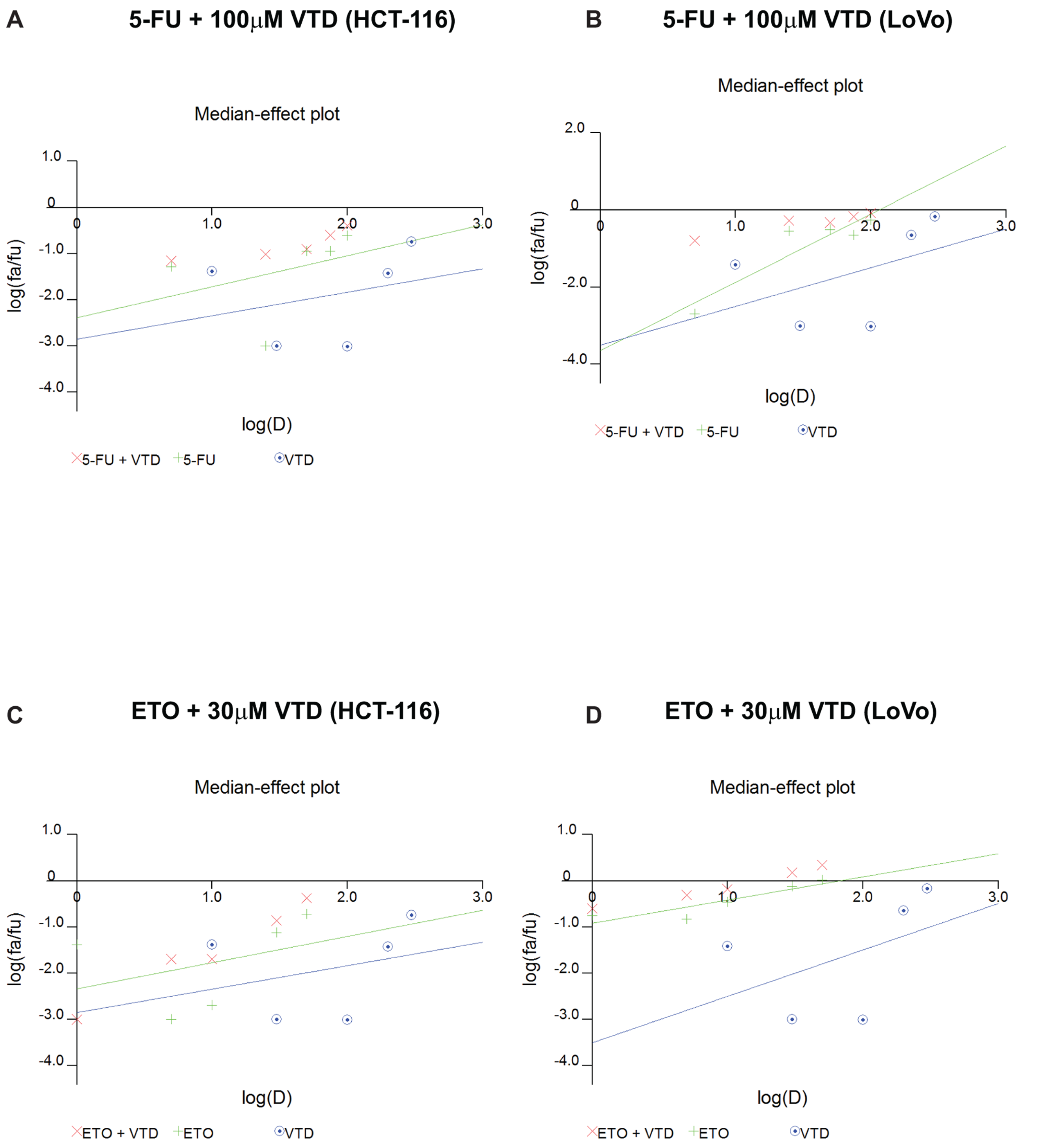


**Supplementary Figure S7: Combination of VTD and standard chemotherapies results in a synergistic suppression of tumor cell viabilities.** HCT-116, poorly differentiated cells, and LoVo, well-differentiated cells, were incubated with 5-FU **A.** and **B.** or etoposide (ETO, **C.** and **D.**) for 72 hours ± VTD (100 µM or 30 µM, respectively). Median-effect plots created by CalcuSyn software determined the synergistic, additive, or antagonistic response of the two drugs when used in combination.

**Supplementary Table S1: Relative fold change of mot-2 and UBXN2A proteins in 48 colon tumor lysates compared with their normal adjacent colon tissues determined by western blot**

| Patient # | Gender | Age | Grade | Stage | TNM | Type | mot-2 (fold changes) | UBXN2A (fold Changes) |
|---|---|---|---|---|---|---|---|---|
| 1 | Male | 66 | 1 | n/a | T4N2M0 | Adenocarcinoma | 0.44 | 0.39 |
| 2 | Female | 70 | 2 | n/a | n/a | Adenocarcinoma | 1.99 | 0.40 |
| 3 | Female | 63 | n/a | III | T3NxM0 | Papillary Adenocarcinoma | 1.07 | 0.54 |
| 4 | Female | 56 | 2 | III | T3N0M0 | Papillary Adenocarcinoma | 1.51 | 0.54 |
| 5 | Male | 54 | n/a | IIIB | T3N1M0 | Adenocarcinoma | 0.40 | 0.54 |
| 6 | Male | 45 | n/a | III | T3N1M0 | Mucinous Adenocarcinoma | 2.09 | 0.64 |
| 7 | Male | 61 | 2 | III | T3NxM0 | Adenocarcinoma | 1.89 | 0.69 |
| 8 | Female | 66 | 1 | IIIA | T2N1M0 | Adenocarcinoma | 2.85 | 0.74 |
| 9 | Female | 73 | 1 | IIIA | T2N1M0 | Mucinous Adenocarcinoma | 0.94 | 0.78 |
| 10 | Female | 53 | 1 | III | T3N1M0 | Mucinous Adenocarcinoma | 1.85 | 0.78 |
| 11 | Male | 46 | n/a | IIB | n/a | Adenocarcinoma | 1.15 | 0.78 |
| 12 | Female | 46 | 1 | IIIB | T3N1M0 | Mucinous Adenocarcinoma | 0.88 | 0.78 |
| 13 | Female | 43 | 1 | IIIB | T3N1M0 | Adenocarcinoma | 2.13 | 0.80 |
| 14 | Male | 50 | 3 | IIIA | T2N1M0 | Adenocarcinoma | 2.63 | 0.81 |
| 15 | Male | 49 | 2 | II | T3N0M0 | Adenocarcinoma | 2.12 | 0.83 |
| 16 | Male | 25 | n/a | n/a | T3N1M0 | Eosinophylic Granuloma | 1.74 | 0.85 |
| 17 | Male | 41 | 1 | II | T3N0M0 | Adenocarcinoma | 1.46 | 0.88 |
| 18 | Male | 66 | 2 | III | T2N1M0 | Adenocarcinoma | 1.79 | 0.93 |
| 19 | Male | 25 | 1 | I | T2N0M0 | Adenocarcinoma | 1.05 | 0.95 |
| 20 | Male | 77 | 2 | I | T2N0M0 | Adenocarcinoma | 2.82 | 0.96 |
| 21 | Male | 67 | 3 | II | T4NxM0 | Adenocarcinoma | 1.71 | 0.96 |
| 22 | Female | 67 | n/a | II | T2N0M0 | Adenocarcinoma | 1.06 | 0.97 |
| 23 | Male | 52 | 3 | II | T3N0M0 | Adenocarcinoma | 2.39 | 0.99 |
| 24 | Male | 44 | n/a | n/a | T3N1M0 | Lymphoma | 1.52 | 1.01 |
| 25 | Female | 38 | 1 | I | T2N0M0 | Adenocarcinoma | 1.19 | 1.02 |
| 26 | Male | 46 | n/a | n/a | n/a | Non-Hodgkin Lymphoma | 1.15 | 1.05 |
| 27 | Male | 40 | n/a | n/a | T3N1M0 | Multipolyposis | 3.44 | 1.09 |
| 28 | Female | 59 | 2 | III | T3N1M0 | Adenocarcinoma | 3.78 | 1.13 |
| 29 | Male | 26 | n/a | IV | T2N1M0 | Mucinous Adenocarcinoma | 1.44 | 1.14 |
| 30 | Female | 74 | 2 | IIIA | T2N1M0 | Adenocarcinoma | 1.58 | 1.14 |
| 31 | Female | 57 | 2 | n/a | T2N0M0 | Adenocarcinoma | 1.57 | 1.16 |
| 32 | Male | 75 | 1 | I | T2N0M0 | Adenocarcinoma | 3.01 | 1.21 |
| 33 | Male | 79 | 1 | IIIB | T3N1M0 | Non-Hodgkin Lymphoma | 2.36 | 1.23 |
| 34 | Male | 55 | 1 | IIIB | T3N1M0 | Mucinous Adenocarcinoma | 1.19 | 1.24 |

(*Continued*)

| Patient # | Gender | Age | Grade | Stage | TNM | Type | mot-2 (fold changes) | UBXN2A (fold Changes) |
|---|---|---|---|---|---|---|---|---|
| 35 | Female | 69 | n/a | IV | n/a | Adenocarcinoma | 1.63 | 1.27 |
| 36 | Female | 70 | 3 | IV | T3NxM1 | Adenocarcinoma | 6.12 | 1.32 |
| 37 | Female | 46 | 1 | II | T3N0M0 | Adenocarcinoma | 3.25 | 1.33 |
| 38 | Male | 79 | 2 | II | T3N0Mx | Adenocarcinoma | 2.64 | 1.37 |
| 39 | Male | 35 | 2 | n/a | T4N3M0 | Adenocarcinoma | 6.95 | 1.43 |
| 40 | Female | 63 | 2 | III | T3NxM0 | Adenocarcinoma | 7.60 | 1.46 |
| 41 | Male | 65 | 2 | I | T2N0M0 | Leiomyosarcoma | 3.71 | 1.49 |
| 42 | Female | 32 | n/a | IIIA | T2N1M0 | Non-Hodgkin Lymphoma | 1.70 | 1.50 |
| 43 | Male | 77 | 1 | II | T2N1M0 | Adenocarcinoma | 1.78 | 1.58 |
| 44 | Male | 68 | 3 | IIIA | T2N1M0 | Adenocarcinoma | 2.91 | 1.92 |
| 45 | Male | 44 | 1 | II | T3N0M0 | Adenocarcinoma | 1.27 | 2.01 |
| 46 | Female | 58 | n/a | IV | T3N1M0 | Adenocarcinoma | 2.77 | 2.20 |
| 47 | Male | 38 | 2 | IIIA | T2N1M0 | Adenocarcinoma | 4.55 | 2.31 |
| 48 | Male | 42 | 2 | III | T3N1M0 | Mucinous Adenocarcinoma | 3.07 | 2.34 |

**Supplementary Table S2: Relative fold change of mot-2 proteins in 55 breast tumor lysates compared with their normal adjacent breast tissues determined by western blot**

| Patient # | Gender | Age | Grade | Stage | TNM | Type | mot-2 (fold changes) |
|---|---|---|---|---|---|---|---|
| 1 | F | 54 | 2 | IIA | T2N0M0 | Ductal Carcinoma | 2.48 |
| 2 | F | 36 | 2 | IIIB | T2N1M0 | Ductal Carcinoma | 5.17 |
| 3 | F | 61 | 2 | IV | T4N2Mx | Ductal Carcinoma | 0.92 |
| 4 | F | 72 | 2 | II | T2N0Mx | Ductal Carcinoma | 1.32 |
| 5 | F | 45 | 2 | IV | T4N2Mx | Ductal Carcinoma | 3.23 |
| 6 | F | 42 | 2 | IIA | T2N0M0 | Ductal Carcinoma | 4.14 |
| 7 | F | 44 | 2 | IIIB | T2N1M0 | Ductal Carcinoma | 1.59 |
| 8 | F | 39 | 2 | IV | T2N1M0 | Ductal Carcinoma | 6.73 |
| 9 | F | 54 | 2 | II | T4bN1M0 | Ductal Carcinoma | 2.97 |
| 10 | F | 54 | 2 | IV | T2N1M0 | Ductal Carcinoma | 4.06 |
| 11 | F | 46 | 2 | IIB | T3N0M0 | Ductal Carcinoma | 2.18 |
| 12 | F | 55 | 2 | IIIB | T4bN2M0 | Ductal Carcinoma | 1.31 |
| 13 | F | 44 | 2 | IIB | T2N1M0 | Ductal Carcinoma | 1.56 |
| 14 | F | 63 | 2 | IIB | T2N1M0 | Ductal Carcinoma | 5.57 |
| 15 | F | 61 | 2 | IIB | T2N1M0 | Ductal Carcinoma | 3.41 |
| 16 | F | 61 | 2 | IIA | T1N1M0 | Ductal Carcinoma | 1.90 |
| 17 | F | 85 | 2 | IV | T4N1Mx | Ductal Carcinoma | 1.27 |

(*Continued*)

| Patient # | Gender | Age | Grade | Stage | TNM | Type | mot-2 (fold changes) |
|---|---|---|---|---|---|---|---|
| 18 | F | 75 | 2 | IV | T4N0M0 | Ductal Carcinoma | 2.12 |
| 19 | F | 47 | 2 | II | T2NxMx | Ductal Carcinoma | 0.99 |
| 20 | F | 43 | 3 | IIA | T2N0M0 | Ductal Carcinoma | 5.29 |
| 21 | F | 61 | 3 | IIB | T2N1M0 | Ductal Carcinoma | 2.42 |
| 22 | F | 39 | 3 | IIB | T2N1M0 | Ductal Carcinoma | 3.09 |
| 23 | F | 35 | 3 | II | T2N1Mx | Ductal Carcinoma | 7.20 |
| 24 | F | 73 | 3 | IIIB | T3N1bMx | Ductal Carcinoma | 2.06 |
| 25 | F | 50 | 3 | IIB | T2N1M0 | Ductal Carcinoma | 6.23 |
| 26 | F | 52 | 3 | IIIA | T3N1M0 | Ductal Carcinoma | 1.62 |
| 27 | F | 49 | 3 | IIIA | T3N1M0 | Ductal Carcinoma | 13.01 |
| 28 | F | 41 | 3 | II | T2NxM0 | Ductal Carcinoma | 5.51 |
| 29 | F | 47 | 3 | IV | T4N1M0 | Ductal Carcinoma | 3.87 |
| 30 | F | 55 | 3 | II | T2N1M0 | Ductal Carcinoma | 3.54 |
| 31 | F | 75 | 3 | II | T2N0M0 | Ductal Carcinoma | 0.61 |
| 32 | F | 53 | 3 | II | T2N1M0 | Ductal Carcinoma | 0.80 |
| 33 | F | 60 | 3 | II | T2N1M0 | Ductal Carcinoma | 0.89 |
| 34 | F | 54 | 3 | IV | T4N2Mx | Ductal Carcinoma | 0.83 |
| 35 | F | 43 | 3 | II | T2N1M0 | Ductal Carcinoma | 1.45 |
| 36 | F | 53 | 3 | II | T2N1Mx | Ductal Carcinoma | 6.43 |
| 37 | F | 43 | 3 | III | T3N2M0 | Ductal Carcinoma | 0.58 |
| 38 | F | 58 | 3 | II | T2N1M0 | Ductal Carcinoma | 0.51 |
| 39 | F | 44 | 3 | III | T3N2M0 | Ductal Carcinoma | 3.05 |
| 40 | F | 48 | 2 | IIIB | T4bN2M0 | Ductal Carcinoma | 4.49 |
| 41 | F | 42 | 2 | IIIA | T3N2M0 | Ductal Carcinoma | 3.36 |
| 42 | F | 32 | 2 | IIB | T2N1M0 | Ductal Carcinoma | 7.91 |
| 43 | F | 41 | 2 | IIA | T2N0M0 | Intraductal Carcinoma | 10.33 |
| 44 | F | 40 | 2 | IIB | T2N1M0 | Intraductal Carcinoma | 8.28 |
| 45 | F | 50 | 2 | IIA | T2N0M0 | Intraductal Carcinoma | 5.79 |
| 46 | F | 51 | 1 | IIB | T2N1M0 | Ductal Carcinoma | 1.85 |
| 47 | F | 47 | 1 | IIIA | T3N1M0 | Ductal Carcinoma | 1.63 |
| 48 | F | 38 | 1 | IIIA | T3N1M0 | Ductal Carcinoma | 6.04 |
| 49 | F | 60 | 1 | IIA | T2N0M0 | Ductal Carcinoma | 3.45 |
| 50 | F | 39 | 1 | IIB | T2N1M0 | Ductal Carcinoma | 1.74 |

(*Continued*)

| Patient # | Gender | Age | Grade | Stage | TNM | Type | mot-2 (fold changes) |
|---|---|---|---|---|---|---|---|
| 51 | F | 43 | 2 | IIIA | T3N1M0 | Mucinous Carcinoma | 1.17 |
| 52 | F | 51 | 1 | II | T2N1Mx | Mucinous Carcinoma | 3.67 |
| 53 | F | 59 | n/a | IB | T2N0Mx | Mucinous Carcinoma | 2.30 |
| 54 | F | 39 | n/a | III | T3N2M0 | Lobular Carcinoma | 2.54 |
| 55 | F | 46 | 3 | IV | T4N1bMx | Metastatic Carcinoma | 1.26 |

**Supplementary Table S3: Details of antibodies, manufacturer and the dilution used for WB and flow-cytometry**

| Name | Manufacturer | Dilution |
|---|---|---|
| Rabbit polyclonal anti-UBXN2A against #C-IQRLQKTASFRELS peptide located in the c-terminus of human UBXN2A (#NM_181713) | Pacific Immunology Corp | 1:1000 (WB) |
| Anti-p53 antibody (DO-1) | Santa Cruz biotechnology | 1:1000 |
| Anti-GRP75 (mot-2) antibody (D-9) | Santa Cruz biotechnology | 1:2000 |
| Anti-NSFL1C antibody (p47) | ABCAM | 1:1000 |
| Anti-HSC70 | Santa Cruz biotechnology | 1:5000 |
| Anti-Caspase-3 | Cell Signaling | 1:200 (WB)<br>1:250 (FC) |
| Anti-actin | Cell signaling | 1:200 |
| Anti-cleaved PARP (Asp214) | Cell signaling | 1:200 (FC) |
| Mouse Anti-Human ORC-2 antibody | BD Biosciences | 1:1000 |
| Mouse anti-Glyceraldehyde-3-Phosphate Dehydrogenase antibodies (anti-GAPDH, loading controls and cytoplasmic marker). | Millipore | 1:20000 |
| IRDye 800CW Goat anti-Rabbit IgG (H+L), | LI-COR Corporate | 1:3000 |
| IRDye 800CW Goat anti-Mouse IgG (H+L), | LI-COR Corporate | 1:3000 |

**Supplementary Table S4: Analysis of a mode of combined treatment with different concentrations of VTD and fixed suboptimal dosage of etoposide (ETO) in HCT-116 (poorly differentiated) and LoVo (well-differentiated) colon cancer cells**

**HCT-116**

| Drugs | | 24 hours | | 48 hours | | 72 hours | |
|---|---|---|---|---|---|---|---|
| VTD (μM) | ETO (μM) | CI | Description | CI | Description | CI | Description |
| 10 | 5 | 1.227 | Moderate antagonism | 33.691 | Very strong antagonism | 37.158 | Very strong antagonism |
| 30 | 5 | 0.743 | Moderate synergism | 38.703 | Very strong antagonism | 49.102 | Very strong antagonism |
| 100 | 5 | 3.926 | Strong antagonism | 56.244 | Very strong antagonism | 10.43 | Very strong antagonism |
| 200 | 5 | 0.13 | Strong Synergism | 0.403 | Synergism | 0.404 | Synergism |
| 300 | 5 | 0.058 | Very strong synergism | 0.182 | Strong synergism | 0.038 | Very strong synergism |

**LoVo**

| Drugs | | 24 hours | | 48 hours | | 72 hours | |
|---|---|---|---|---|---|---|---|
| VTD (μM) | ETO (μM) | CI | Description | CI | Description | CI | Description |
| 10 | 5 | 2.311 | Antagonism | 8.2 | Strong antagonism | 318.722 | Very strong antagonism |
| 30 | 5 | 2.463 | Antagonism | 12.6 | Very strong antagonism | 12.635 | Very strong antagonism |
| 100 | 5 | 0.88 | Slight synergism | 0.883 | Slight synergism | 0.721 | Moderate synergism |
| 200 | 5 | 0.084 | Very strong synergism | 0.27 | Strong synergism | 0.205 | Strong synergism |
| 300 | 5 | 0.06 | Very strong synergism | 0.188 | Strong synergism | 0.073 | Very strong synergism |

CI (combination index) values were generated from the data of Figure 6A–6F (average of three measurements) and applied as cell death value to calculate median effect plot curves for each treatment using CalcuSyn, version 2.0 software. CI >1 denotes antagonism, CI = 1 denotes additivity, and CI < 1 denotes synergism.

**Supplementary Table S5: Analysis of a mode of combined treatment with VTD+ 5-FU or VTD + etoposide (ETO) in HCT-116 (poorly differentiated) and LoVo (well-differentiated) colon cancer cells**

| HCT-116 (5-FU +VTD) | | | |
|---|---|---|---|
| 5-FU (µM) | VTD (µM) | CI | Description |
| 5 | 100 | 0.116 | Strong Synergism |
| 25 | 100 | 0.249 | Strong Synergism |
| 50 | 100 | 0.325 | Synergism |
| 75 | 100 | 0.165 | Strong Synergism |
| 100 | 100 | 0.109 | Strong Synergism |

| HCT-116 (ETO + VTD) | | | |
|---|---|---|---|
| ETO (µM) | VTD (µM) | CI | Description |
| 1 | 30 | 73.954 | Very strong antagonism |
| 5 | 30 | 0.516 | Synergism |
| 10 | 30 | 0.877 | Slight Synergism |
| 30 | 30 | 0.079 | Very strong synergism |
| 50 | 30 | 0.017 | Very strong synergism |

| LoVo (5-FU + VTD) | | | |
|---|---|---|---|
| 5-FU (µM) | VTD (µM) | CI | Description |
| 5 | 100 | 0.32 | Synergism |
| 25 | 100 | 0.368 | Synergism |
| 50 | 100 | 0.73 | Moderate synergism |
| 75 | 100 | 0.86 | Slight synergism |
| 100 | 100 | 1.004 | Nearly Additive |

| LoVo (ETO + VTD) | | | |
|---|---|---|---|
| ETO (µM) | VTD (µM) | CI | Description |
| 1 | 30 | 0.275 | Strong synergism |
| 5 | 30 | 0.324 | Synergism |
| 10 | 30 | 0.347 | Synergism |
| 30 | 30 | 0.204 | Strong synergism |
| 50 | 30 | 0.154 | Strong synergism |

CI (combination index) values were generated from the data of Figure 6I–6L (average of three measurements) and applied as cell death value to calculate median effect plot curves for each treatment using CalcuSyn, version 2.0 software. CI >1 denotes antagonism, CI = 1 denotes additivity, and CI < 1 denotes synergism.

## REFERENCES


1. Brotherick I, Shenton BK, Cowan WK, Angus B, Horne CH, Higgs MJ, Lennard TW. p53 expression measured by flow cytometry. A comparison of three monoclonal antibodies and the relationship with grade and DNA ploidy in breast cancer. Cancer Immunol Immunother. 1995; 41:146–150.
2. Ermak G, Cancasci VJ, Davies KJ. Cytotoxic effect of doxycycline and its implications for tet-on gene expression systems. Anal Biochem. 2003; 318:152–154.
3. Zhang J, Wang C, Ke N, Bliesath J, Chionis J, He QS, Li QX, Chatterton JE, Wong-Staal F, Zhou D. A more efficient RNAi inducible system for tight regulation of gene expression in mammalian cells and xenograft animals. RNA. 2007; 13:1375–1383.
4. Hensley H, Devarajan K, Johnson JR, Piwnica-Worms D, Godwin AK, von Mehren M, Rink L. Evaluating new therapies in gastrointestinal stromal tumor using *in vivo* molecular optical imaging. Cancer Biol Ther. 2014; 15:911–918.
5. Chu C, Huang X, Chen CT, Zhao Y, Luo JJ, Gray BD, Pak KY, Dun NJ. *In vivo* imaging of brain infarct with the novel fluorescent probe PSVue 794 in a rat middle cerebral artery occlusion-reperfusion model. Mol Imaging. 2013; 12:8–16.
6. Morton CL, Houghton PJ. Establishment of human tumor xenografts in immunodeficient mice. Nat Protoc. 2007; 2:247–250.
7. Maaser C, Schoeppner S, Kucharzik T, Kraft M, Schoenherr E, Domschke W, Luegering N. Colonic epithelial cells induce endothelial cell expression of ICAM-1 and VCAM-1 by a NF-kappaB-dependent mechanism. Clin Exp Immunol. 2001; 124:208–213.
8. Bijnsdorp IV, Giovannetti E, Peters GJ. Analysis of drug interactions. Methods Mol Biol. 2011; 731:421–434.
